# Do promotions of healthier or more sustainable foods increase sales? Findings from three natural experiments in UK supermarkets

**DOI:** 10.1186/s12889-024-19080-x

**Published:** 2024-06-21

**Authors:** Madison Luick, Lauren Bandy, Carmen Piernas, Susan A. Jebb, Rachel Pechey

**Affiliations:** 1 Nuffield Department of Primary Care Health Sciences, Radcliffe Observatory Quarter, University of Oxford, Oxford, OX2 6GG UK; 2 Department of Biochemistry and Molecular Biology II, Institute of Nutrition and Food Technology (INYTA), Center for Biomedical Research (CIBM), Biosanitary Research Institute (IBS), University of Granada, Granada, Spain

**Keywords:** Supermarket, Promotions, UK, Food, Health, Sustainability

## Abstract

**Background:**

Dietary changes are necessary to improve population health and meet environmental sustainability targets. Here we analyse the impact of promotional activities implemented in UK supermarkets on purchases of healthier and more sustainable foods.

**Methods:**

Three natural experiments examined the impact of promotional activities on sales of a) no-added-sugar (NAS) plant-based milk (in 199 stores), b) products promoted during ‘Veganuary’ (in 96 stores), and c) seasonal fruit (in 100 non-randomised intervention and 100 matched control stores). Data were provided on store-level product sales, in units sold and monetary value (£), aggregated weekly. Predominant socioeconomic position (SEP) of the store population was provided by the retailer. Analyses used interrupted time series and multivariable hierarchical mixed-effects models.

**Results:**

Sales of both promoted and total NAS plant-based milks increased significantly during the promotional period (Promoted:+126 units, 95%CI: 105–148; Overall:+307 units, 95%CI: 264–349). The increase was greater in stores with predominately low SEP shoppers. During Veganuary, sales increased significantly for plant-based foods on promotion (+60 units, 95%CI: 37–84), but not for sales of plant-based foods overall (dairy alternatives: -1131 units, 95%CI: -5821–3559; meat alternatives: 1403 units, 95%CI: -749–3554). There was no evidence of a change in weekly sales of promoted seasonal fruit products (assessed via ratio change in units sold: 0.01, 95%CI: 0.00–0.02), and overall fruit category sales slightly decreased in intervention stores relative to control (ratio change in units sold: -0.01, 95%CI: -0.01–0.00).

**Conclusion:**

During promotional campaigns there was evidence that sales of plant-based products increased, but not seasonal fruits. There was no evidence for any sustained change beyond the intervention period.

**Supplementary Information:**

The online version contains supplementary material available at 10.1186/s12889-024-19080-x.

## Background

Poor diets, high in saturated fats, sugars, and salt and low in fruits and vegetables, increase the population burden of non-communicable diseases [1]. Yet, while much of the world still does not have access to sufficient nutritious foods, global food production is also pushing the constraints of what is environmentally sustainable [2]. In the UK dietary habits are gradually moving towards healthier and more sustainable diets though the pace of change is slow. For example, average individual daily meat consumption of red, white, and processed meat in the UK decreased from 103.7 g to 83.6 g between 2008 to 2019 [3] and fruit and vegetable consumption has increased since early 2000s, from a mean 3.3 to 3.5 portions a day for men, and 3.5 to 3.8 portions a day for women [4]. With pressing population health and climate concerns, interventions are needed to accelerate these trends to improve the health of people and the planet.

Food purchasing is a key determinant of food intake. In economically developed countries the majority of food is purchased in grocery stores—in the US, at least 2/3rd of household energy purchases come from large supermarkets [5], while an estimated 78% of expenditure on food and drink in the UK is spent on household food and drink [6]. This presents an opportunity to shape dietary habits. Systematic reviews have identified that changing product positioning (e.g. moving or removing products from ends of aisles or near tills), adding or removing price promotions, increasing availability, and displaying messaging about products can influence food purchasing in supermarkets [7–10]. For example, the removal of confectionery from prominent positions in stores resulted in a decrease in purchasing [11], and increasing the availability or more prominent positioning and promotion of healthier foods may result in an increase in purchasing of foods that are more beneficial for health [12–14]. While the Government in England has introduced legislation so that products classified as less healthy (based on a nutrient profiling model) cannot be placed near checkout tills, store entrances, or end-of-aisle areas [15], it is as yet unclear how effective implementation and adherence to the policy has been. Regardless, further measures will be needed, and retailers continue to have the ability to make other changes on a voluntary basis as part of their commitments to health and sustainability.

Policymakers need to be able to identify strategies that are effective at increasing the healthiness and sustainability of food purchases, but the evidence base is limited. Data from large-scale studies in real supermarket settings are scarce because of the complexity of securing collaborations with retailers [16–18]. Evidence is particularly limited for strategies to encourage more sustainable diets, as previous studies have tended to focus on stimulating purchases of healthier foods (although these may also have co-benefits for the environment [19, 20]).

It is also important that interventions do not exacerbate inequalities. People in lower socioeconomic groups tend to purchase less healthy foods overall [21–23] and consumers that shop at low- versus high-price supermarkets tend to purchase fewer fruits and vegetables [24]. Through lowering prices, promotions could benefit these groups. However, there has also been evidence to suggest that those in higher socioeconomic groups are more responsive to price promotions, possibly as a result of greater purchasing power and ability to take advantage of a price promotion when it is in place [25]. Given existing health inequalities, the aim is to identify interventions that work best for those who may benefit most from healthier diets.

Here we analyse in-store promotional activity interventions developed and implemented by a leading UK retailer, using these natural experiments to assess both the overall impact of interventions on sales of promoted products and the broader category, and examine the effect of interventions in stores with customers predominately of higher or lower socioeconomic position (SEP). The aim of this paper was to consider how interventions intended to encourage purchasing of products considered healthier or more sustainable could influence purchasing patterns.

## Methods

### Study design and data source

In cooperation with the Consumer Goods Forum (CGF) Collaboration for Healthier Lives (CHL, https://www.theconsumergoodsforum.com/health-wellness/healthier-lives/), data was collected from one major UK retailer, who tested three sets of promotional activity in 2021 with the aim of encouraging healthier and more sustainable purchases. The retail partner developed the promotions and implemented them in stores. Each intervention varied in duration, extent of promotional activity and number of stores, but all three involved price promotion and two involved prominent positioning interventions. This study tested the impact of the combined promotional activity (described below), as it is not possible to isolate the impact of any one promotional strategy. Data was made available to researchers through cooperation with CGF as part of an independent evaluation of these trials.

Promoted products (determined by the retailer) for each of the three natural experiments included the following products: (experiment 1) no added sugar (NAS) plant-based milks, (experiment 2) vegan products during a vegan January event (i.e. Veganuary), and (experiment 3) seasonal fruits and vegetables. These cover a range of possible healthy and/or sustainable foods products that could be promoted, albeit by no means a complete list.

Aggregate data was provided to the independent evaluation team on sales of selected categories (units and value) from 2018 to 2021. The protocol was pre-registered at https://osf.io/9rkde/. R 4.1.3 was used for all statistical analyses.

### Interventions and analyses

#### No added sugar (NAS) plant-based milks

##### Intervention

The NAS plant-based milk intervention ran for three weeks in May 2021 and involved price promotions, banners, aisle fins and social media encouraging the purchasing of a brand of NAS plant-based milk. Price reduction stickers were placed near the products, coupons were located near the checkout till, and online there were banners for the promoted products during the period of promotion. Positioning of milks remained unchanged under this promotion. The price promotion involved offering selected NAS plant-based milks, most commonly priced from £1.30 to £1.70, for £1 during the promotional period. This included eight different NAS plant-based milk products offered on promotion (with one store offering only 7). The intervention was applied in all stores, with the retailer providing data from a sample of 199 stores across the UK representing a range of customer demographics and regions for analysis, based on in-house classifications for store affluence, store size, and region.

##### Data

Sales data, units and value (£), of promoted plant-based milks and all plant-based milks, aggregated at the weekly level, were used as primary outcomes. To analyse the impact on all NAS plant-based milks, those milks were identified based on terms ‘no added sugar’, ‘unsweetened’, and ‘light’ in the product name. Nutritional information for those with ‘light’ in the name was checked and inclusion in analyses was limited to only those that had no added sugars. This totalled 30 unique NAS plant-based milk products, with stores offering between 19 and 29 of these products during the study period, commonly priced between £1 and £1.70. We also analysed sales of all plant-based milk products, of which there were 130, with stores offering between 57 and 125 different products over the study period, and products commonly priced between £1 and £1.70.

Demographic data available included retailer-defined classifications for the predominant age and household structure of shoppers in store (“store age”) and included middle aged adult, young adult, retired, families with pre-school children, families with school children, and elderly. For purposes of analysis, retired and elderly were combined into one group. Families with pre-school children and the families with school children were also grouped. Store affluence, was classified as price sensitive, mid-market, upmarket, and super-upmarket, interpreted as increasing SEP of the predominant shoppers. Postcode data was also provided, which allowed for analysis by Index of Multiple Deprivation (IMD), available from the UK government website providing data from 2019 [26]. For analysis, IMD deciles were grouped into the following categories: Low IMD (deciles 1–3; most deprived), Medium IMD (deciles 4–7), High IMD (deciles 8–10; least deprived).

##### Analysis

The primary method of analysis for all three trials was interrupted time-series (ITS). Newey-West standard errors with lag 4 were applied, determined prior to analysis following similar previous studies [11, 13]. This was to account for autocorrelation and heteroskedasticity in the data, and was set prior to analyses so that there would be consistency across all trials. ITS models were run on data up to the end of the intervention, with the interruption occurring at the start of the intervention and running into the fourth week. Multivariable hierarchical mixed-effects models were used to adjust for demographic characteristics. For the mixed-effects models, the full time period of available data up until the end of the intervention was used, but each model included a term for the intervention time period. Where sales value (in £) was the outcome, linear mixed-effects models were used, and where units sold was the outcome, negative binomial models were used with results reported as an incidence rate ratio (IRR). Where the intervention was shown to be significant, an additional mixed-effects model, either linear mixed-effects or negative binomial depending on the outcome being tested, was run including an interaction term between affluence and intervention.

#### Veganuary

##### Intervention

The Veganuary intervention ran for four weeks in January 2021. Price promotions, banners and social media were deployed in store to encourage purchasing of plant-based foods across a range of food categories. The retailer also utilised television advertisements, radio partnerships, influencers on social media, email, and online advertisements to further draw attention to the ongoing promotion. In stores, there were smart screens with information about the promotion, recipe cards, recommendations next to vegan products of how they could be used, and large signs hanging overhead to increase visibility of the promotion. Promoted items were placed at the end of aisles, otherwise product positioning was not targeted. Promotions included price reductions mostly ranging from £0.20 to £1, with a sticker highlighting the price reduction and an edge of shelf placard drawing attention to the Veganuary promotional campaign. During Veganuary, most promoted products cost between £1 and £2, with an average price of £1.73, compared to a base price range from £1.6 to £2.75, with an average price of £2.29. The intervention was applied in all retailer stores, with analysis focused a priori on 96 larger stores which implemented the most promotional activity, for example using smart screens to advertise the promotions in store and including substantial end-of-aisle activity to promote products.

##### Data

Primary outcome measures for analysis were weekly sales data, units and value (£), for the foods promoted in the Veganuary intervention. Promoted foods were considered to be plant-based food products and alternatives which offered a direct substitute for animal-based products. For example, plant-based ready meals, plant-based meat, non-dairy milks, etc. We did not include products such as canned vegetables that were also promoted under this campaign to boost vegetable intake. Wider categories were also analysed where compensatory behaviours may be anticipated. For example, meat alternative products were promoted as part of Veganuary, so it was hypothesised that effects could be seen in the wider meat and meat alternative categories as purchasing behaviours shifted. Meat and meat alternatives were classified based on buyer area categories provided by the retailer. Dairy and dairy alternatives were classified based on key retailer-provided categories, supplemented by identifying products and brands through keywords related to the appropriate category (See Appendix for list of identifying terms). For this analysis, data was provided from January 2018 to December 2021. However, due to data limitations in product labelling, data before April 2019 could not be used when product names were required for keyword searches to determine identification (i.e. dairy/dairy alternative and ready meal analyses). Demographic data on store age and store affluence were provided by the retailer, with the same groupings as for NAS plant-based milk, but with no postcode data.

##### Analysis

ITS analysis with lag 4 was used for primary analysis. Regular peaks and valleys were noted in the Veganuary data, so dummy variables for the month of January and the last week of the year were applied to the model. Following the intervention period, additional data were made available to enable ITS models to be run from April 2019 to December 2021, with the interruption set at the start of the intervention in January 2021. To adjust for demographic characteristics, multivariable hierarchical mixed-effects models were also applied. The full time period of available data up until the end of the intervention was used, but each model included a term for the intervention time period. Where sales value (in £) was the outcome, linear mixed-effects models were used, and where units sold was the outcome, negative binomial models were used with results reported as an incidence rate ratio (IRR). Where the intervention was shown to be significant, an additional mixed-effects model, either linear mixed-effects or negative binomial depending on the outcome being tested, was run including an interaction term between affluence and intervention.

#### Seasonal fruit

##### Intervention

The seasonal fruit intervention ran for approximately 13 weeks, from May to August 2021, with price promotions, tastings and messaging to encourage purchase of seasonal summer fruits. There were 18 different fruit products and one variety of lettuce offered, with all stores in the trial offering all 19 products. Price promotion stickers were prominently placed next to the products on the shelf, and hanging placards were used to draw attention to the aisle location where the promotion was taking place. The price promotions included: multibuy deals (e.g. 2 for £3) that allowed for a mix and match of products, boxes of fruit for a discounted price (usually £3 or £4), or products offered with a fixed 25% discount. During the study period, there was some change in the products included in the price promotion, however, all products were included in the prominent location at the end of the aisle and in the placards drawing attention to the products. The intervention was applied in 100 stores selected by the retailer based on capacity to implement the interventions, with an equivalent number of stores as the control group, matched using proprietary analytics based on customer demographics, store size, stock, and sales.

##### Data

Sales data, units and value (£), of promoted summer fruits and all fresh fruits (i.e. all fruit offered in the store throughout the study period) were used as primary outcomes, aggregated at the weekly level. Demographic data on store age and store affluence were provided by the retailer, with the same groupings as for NAS plant-based milk, but with no postcode data.

ITS analysis was used with Newey-West standard errors and lag 4 applied. Two approaches (difference-in-difference and ratio) were explored to assess how intervention stores compared to control before and during the intervention. A t-test was used to compare the units sold and revenue (in £) between control and intervention store groups. Given the discrepancy in sales between intervention and control stores (with sales in intervention stores approximately double those in control stores), the ratio method was prioritised. ITS models were run on data up to the end of the intervention, with an interruption occurring at the start of the intervention.

##### Analysis

Multivariable hierarchical mixed-effects models were used to adjust for demographic characteristics and analyse differential effects by demographics, with linear mixed models (for sales value in £) and negative binomial models (for sales value in units). Results from negative binomial models were reported as the incidence rate ratio (IRR) or the percentage change from this value. The mixed model compared intervention and control stores during the intervention period, with a fixed effect adjustment, which took an average of the weekly sales (in £ or units depending on the model), for the pre-intervention baseline period.

## Results

Three trials of varying lengths were assessed in this natural experiment, which analysed the impact of promotional activities to encourage the purchase and consumption of healthier or more sustainable foods. There was some overlap in stores used for the experiments. Of the stores that were selected for these three natural experiments, 22 stores were used in all three experiments, 51 stores were used in both the seasonal fruit and Veganuary experiments, 78 were used in both the seasonal fruit and plant-based milk experiments, and 33 were used for both the plant-based milk and Veganuary experiments. Tables 1 and 2 show the full descriptive statistics for the three trials.

**Table 1:** Descriptive statistics on the demographics of the stores included in the three trials

**Trial**	**Demographic Information**	**Category**	**Count**		**Percentage**	
Plant-based milk	Store Age	Middle-aged adult	109		54.77%	
		Young adult	58		29.15%	
		Retired or elderly	5		2.51%	
		Families	27		13.57%	
	Store Affluence Level	Price Sensitive	43		21.61%	
		Mid-Market	114		57.29%	
		Upmarket	22		11.06%	
		Super Upmarket	15		7.54%	
	Index of Multiple Deprivation (IMD)	IMD 1–3	81		40.70%	
		IMD 4–7	69		34.67%	
		IMD 8–10	49		24.62%	
Veganuary	Store Age	Middle-aged adult	51		53.13%	
		Young adult	29		30.21%	
		Retired or elderly	2		2.08%	
		Families	14		14.58%	
	Store Affluence Level	Price Sensitive	16		16.67%	
		Mid-Market	64		66.67%	
		Upmarket	10		10.42%	
		Super Upmarket	6		6.25%	
Seasonal Fruit			**Control**	**Intervention**	**Control**	**Intervention**
	Store Age	Middle-aged adult	48	52	48.00%	52.00%
		Young adult	27	26	27.00%	26.00%
		Retired or elderly	2	2	2.00%	2.00%
		Families	23	20	23.00%	20.00%
	Store Affluence Level	Price Sensitive	16	17	16.00%	17.00%
		Mid-Market	61	53	61.00%	53.00%
		Upmarket	15	21	15.00%	21.00%
		Super Upmarket	8	9	8.00%	9.00%

**Table 2:** Descriptive statistics of the three trials, where pre-intervention is prior to the intervention being implemented, intervention includes the period of the trial, and post-intervention includes the time after the intervention. See methods for how data was utilised in analysis and models

**Trial**	**Data**	**Value**	**Pre-intervention**			**Intervention**			**Post-intervention**		
Plant-based milk		Number of weeks	144			4			27		
		Number of stores	199			199			199		
	Promoted products	Mean (sd) units sold weekly (per store)	66 (56)			142 (87)			63 (46)		
		Mean (sd) weekly revenue (in £) from products (per store)	84 (64)			148 (88)			85 (56)		
	All low or NAS plant-based milk	Mean (sd) units sold weekly (per store)	540 (275)			746 (335)			578 (256)		
		Mean (sd) weekly revenue (in £) from products (per store)	640 (321)			816 (365)			689 (298)		
Veganuary		Number of weeks	156			4			49		
		Number of stores	96			96			96		
	Promoted products	Mean (sd) units sold weekly (per store)	91 (60)			286 (118)			155 (62)		
		Mean (sd) weekly revenue (in £) from products (per store)	167 (123)			597 (255)			323 (133)		
	Whole category (plant-based dairy)	Mean (sd) units sold weekly (per store)	484 (266)			735 (267)			587 (242)		
		Mean (sd) weekly revenue (in £) from products (per store)	634 (368)			1032 (385)			866 (353)		
	Whole category (dairy)	Mean (sd) units sold weekly (per store)	12,066 (4050)			11,093 (3502)			11,139 (3710)		
		Mean (sd) weekly revenue (in £) from products (per store)	18,303 (6350)			18,237 (5932)			18,738 (6364)		
	Whole category (plant-based meat)	Mean (sd) units sold weekly (per store)	313 (118)			337 (126)			315 (113)		
		Mean (sd) weekly revenue (in £) from products (per store)	658 (250)			723 (272)			688 (245)		
	Whole category (meat)	Mean (sd) units sold weekly (per store)	20,348 (7475)			20,038 (7475)			18,379 (6394)		
		Mean (sd) weekly revenue (in £) from products (per store)	54,612 (20,834)			51,085 (18,523)			46,490 (16,173)		
Seasonal Fruit			**Control**	**Intervention**	**t-test**^a^	**Control**	**Intervention**	**t-test**^a^	**Control**	**Intervention**	**t-test**^a^
		Number of weeks	147	147	N/A	13	13	N/A	19	19	N/A
		Number of stores	100	100	N/A	100	100	N/A	100	100	N/A
	Promoted products	Mean (sd) units sold weekly (per store)	2577 (1229)	5101 (2414)	< 0.0001	3853 (1492)	7624 (2728)	< 0.0001	1831 (730)	3640 (1465)	< 0.0001
		Mean (sd) weekly revenue (in £) from products (per store)	4790 (2192)	9467 (4307)	< 0.0001	6,669 (2421)	13,185 (4415)	< 0.0001	3343 (1391)	6633 (2793)	< 0.0001
	Whole category	Mean (sd) units sold weekly (per store)	1,792,175 (606,654)	1,708,429 (612,040)	< 0.0001	1,795,639 (548,216)	1,723,418 (568,915)	0.001	1,656,771 (546,550)	1,577,403 (546,999)	< 0.0001
		Mean (sd) weekly revenue (in £) from products (per store)	2,428,995 (843,320)	2,322,546 (850,885)	< 0.0001	2,472,118 (768,211)	2,380,310 (786,571)	0.003	2,198,604 (742,858)	2,100,833 (743,388)	< 0.0001

^a^t-test is between control and intervention store samples for the seasonal fruit intervention

### Plant-based milk intervention

#### Promoted products

Units of promoted plant-based milk sold were 190% higher (a 126-unit increase, 95% CI: 105–148; Supplementary Table 1) on average per store per week during the promotional period, compared to the previous period (66 units on average). A negative binomial model also suggested a significant effect (IRR showing increase of 1.60 times, 95% CI: 1.51–1.69; Supplementary Table 3). However, this uplift was short-lived, with sales returning to baseline after the intervention. See Supplementary Tables 1–3.

#### Whole category

Units sold of all low or NAS plant-based milks were 60% higher (increased by 307 units, 95% CI: 264–349; Supplementary Table 1) per store per week during the promotional period, compared to the previous period. This uplift was again short-lived.

**Fig. 1:**
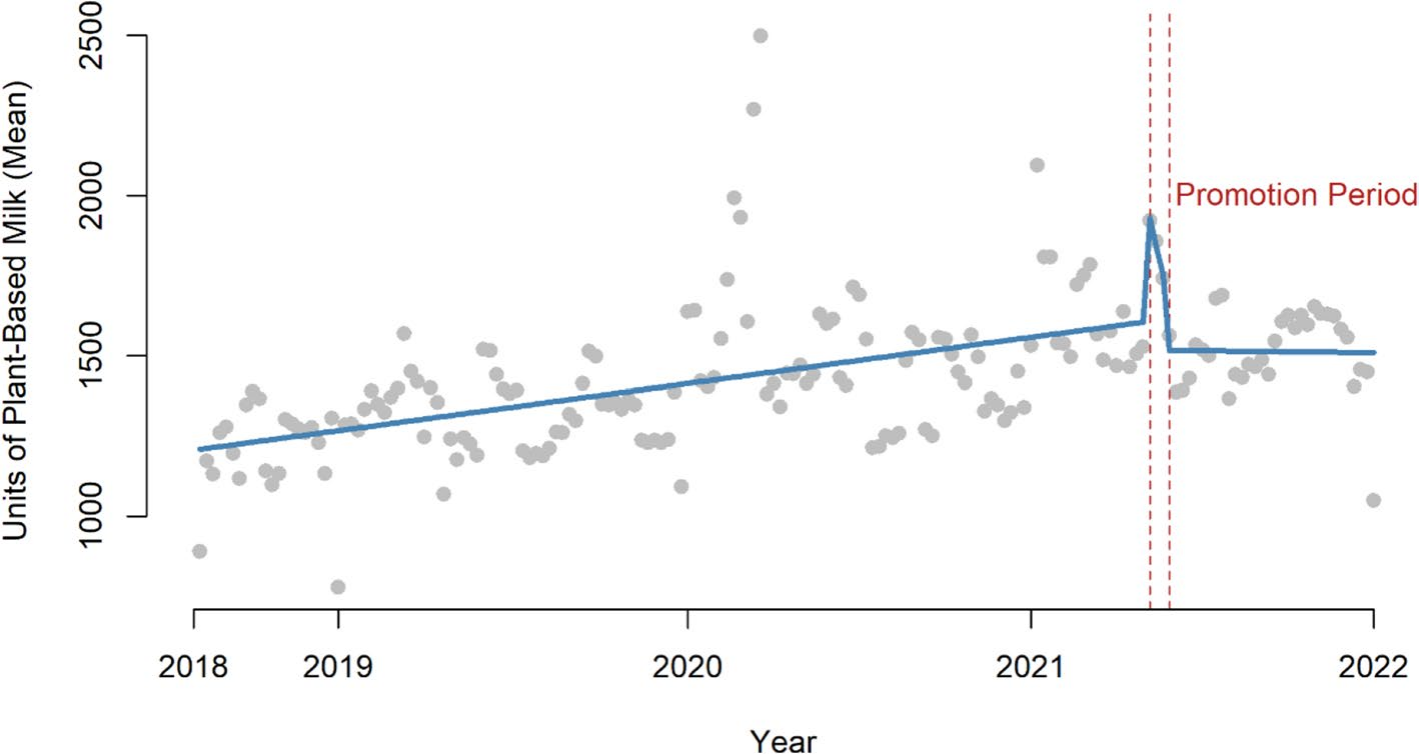
Impact of promotional activity on all plant-based milks, showing a similar trend as that seen in promoted plant-based milks and all NAS plant-based milks (see Supplementary Tables 3–6). This figure shows an analysis with two intervention break points (the start and end of the intervention), as well as the plot of the data after the intervention, although this data was not included in ITS analyses

We also examined all plant-based milks, given the potential environmental benefit. Units of all plant-based milk sold were 30% higher (increased by 463 units, 95% CI: 353–572; Supplementary Table 1) per store per week during the promotional period, compared to the previous period. The effect did not seem to persist (Fig. 1).

#### Plant-based milk demographic analyses

Compared to mid-market stores, the Price Sensitive group of stores sold significantly fewer units of NAS plant-based milk (32% less, 95% CI: 22–41%), and the same was seen for all plant-based milks (30% less, 95% CI: 18%—40%) (Supplementary Table 3, results in text reported as percentage change from IRR). There was no difference between higher SEP stores and mid-market.

**Fig. 2:**
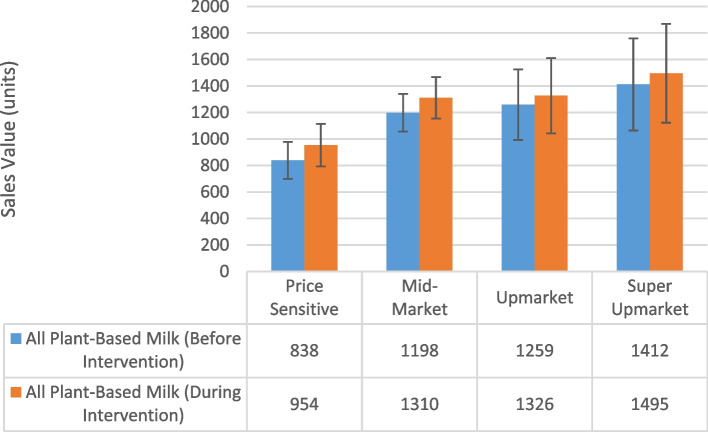
Marginal means by affluence group before and during the intervention

There was a larger intervention effect in lower affluence stores compared to those of higher SEP (Price Sensitive: 8% increase in unit sales, 95% CI: 4–11%; Upmarket: 7% less, 95% CI: 3–10%; Super Upmarket: 10% less, 95% CI: 6–15%). Similar results were found for purchasing of all plant-based milks, although the comparison with Super Upmarket stores no longer reached significance (Price Sensitive: 4% increase unit sales, 95% CI: 1–7%; Upmarket: 4% less, 95% CI: < 0–7%; Super Upmarket: 3% less, 95% CI: 7% less to 1% more) (Fig. 2).

Additional analyses by IMD for plant-based milks (Supplementary Table 4), largely reflected the pattern seen for the affluence variable. Sales of NAS plant-based milks (27% less unit sales, 95% CI: 16–37%) and all plant-based milks (26% less unit sales, 95% CI: 14–36%) were lower in low IMD stores. The intervention effect was less for High IMD (4% less unit sales, 95% CI: 1–7%) and more for Low IMD (7% more unit sales, 95% CI: 4–10%) compared to the reference group of Medium IMD for purchasing of NAS plant-based milks. For purchasing of all plant-based milks, the additional effect of the intervention was again greater in the Low IMD group (5% more unit sales, 95% CI: 2–8%).

### Veganuary intervention: plant-based food options

#### Promoted products

**Fig. 3:**
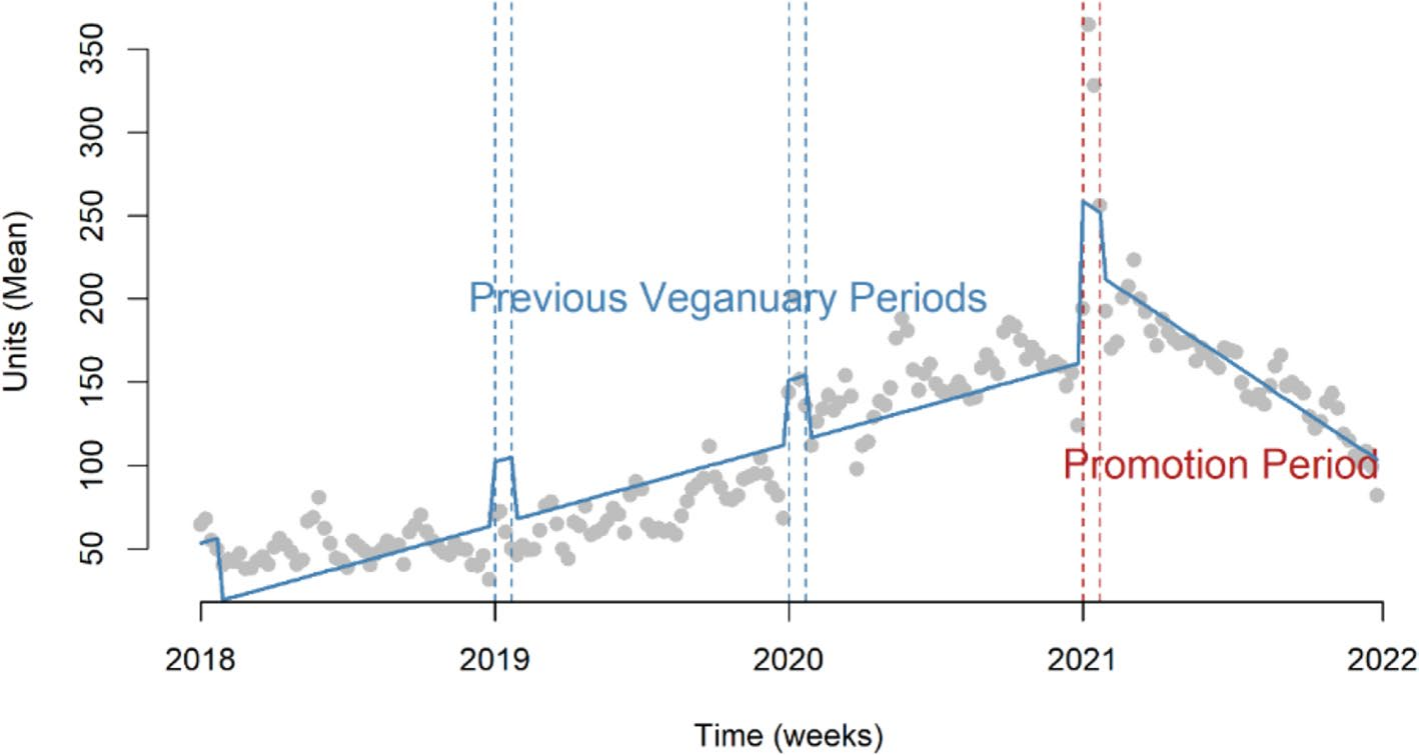
Impact of promotional activity on promoted products in Veganuary

Analyses suggested an increase of 60 units (95% CI: 37–84) of promoted plant-based products being sold on average per store per week during the intervention month (Fig. 3). A negative binomial model also showed a significant increase IRR 1.56 (95% CI: 1.51–1.61) (Supplementary Table 5). Both unit and value sales of promoted products declined after the Veganuary 2021 intervention (Fig. 3).

#### Whole category

ITS analyses found no evidence that the wider sales of plant-based alternatives increased relative to sales of meat and dairy during the specific promotions associated with Veganuary 2021 (See Supplementary Table 6–8).

We conducted exploratory analyses of food categories that were expected to change during Veganuary. Results were mixed, showing small effects and no clear evidence of changes in the expected direction (plant-based dairy alternatives unit sales change: -1131, 95% CI: -5821 –3559; plant-based meat alternatives unit sales change: 1403, 95% CI: -749–3554). There was a significant increase in dairy products during this time (dairy unit sales change: 93,342, 95% CI: 27,953–158,732) but no change in meat unit sales (65,704, 95% CI: -94,261–225,669).

Negative binomial models (Supplementary Table 8) showed that unit sales of meat alternatives increased 1.02 times during the intervention (95% CI: 1.00–1.04), while unit sales of meat were unchanged during the same period (0.98, 95% CI: 0.96 –1.01). Dairy alternatives and dairy unit sales both decreased, with dairy alternatives only 0.85 (95% CI: 0.83–0.88) times the pre-intervention period, and dairy unit sales 0.98 (95% CI: 0.97–0.99) the pre-intervention period. Although prior to Veganuary 2021 there was an increasing trend in sales, sales of all products, both plant-based and non-plant-based, declined after the intervention month.

#### Veganuary demographic analyses

In general, compared to mid-market stores, Price Sensitive stores typically sold less of each kind of product (Promoted Veganuary: 31% less, 95% CI: 18–42%; Dairy Alternatives: 33% less, 95% CI: 19–44%, Dairy: 24% less, 95% CI: 9–36%; Meat alternatives: 27% less, 95% CI: 13–39%; Meat: 17% less, 95% CI: 0–31%; Supplementary Table 8) while Upmarket and Super Upmarket stores sold more of the promoted Veganuary products (Upmarket: 28% more, 95% CI: 4–57%; Super Upmarket: 51% more; 95% CI: 16–97%) and Super Upmarket stores sold more plant-based dairy alternatives (27% more, 95% CI: 2–82%) (Supplementary Table 8).

**Fig. 4:**
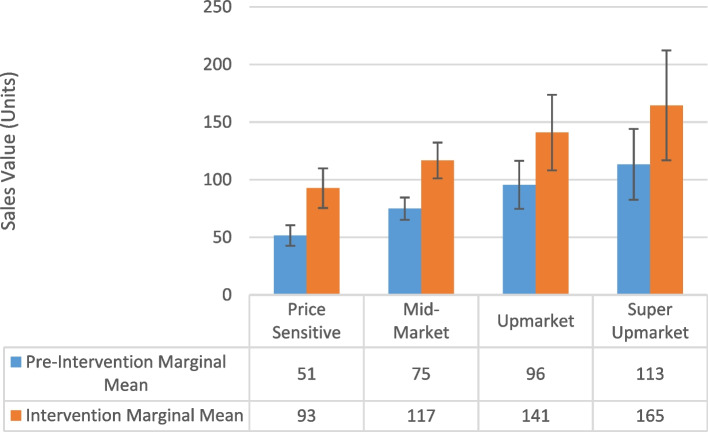
Marginal means by affluence group for promoted Veganuary products

The Veganuary intervention had a greater impact for Price Sensitive stores on sales of promoted Veganuary products (15% more, 95% CI: 7–24%) (Fig. 4), plant-based dairy alternatives (7% more, 95% CI: 1–13%), and dairy products (3% more, 95% CI: > 0%—5% more) when compared to Mid-Market stores (Supplementary Table 8).

In contrast, sales of meat alternatives increased to a greater extent for Super Upmarket and Upmarket stores (Super Upmarket: 11% more, 95% CI: 4–18%; Upmarket: 9% more, 95% CI: 3–15%), with meat having no significant interaction effects (Supplementary Table 8).

### Seasonal fruit intervention

#### Promoted products

There was no clear evidence of an impact on sales of seasonal fruit in control stores compared to intervention stores during the promotional period (change in ratio of units: 0.01, 95% CI: 0.00–0.01, *p* = 0.07) (Supplementary Tables 9–10).

In contrast, a mixed model, conducted using data from each store separately, the incidence rate ratio (IRR) suggested that intervention stores had 1.16 times the units sold per store per week (95%CI: 1.10–1.23), compared to control stores, during the intervention period (and controlling for baseline sales).

#### Whole category

**Fig. 5:**
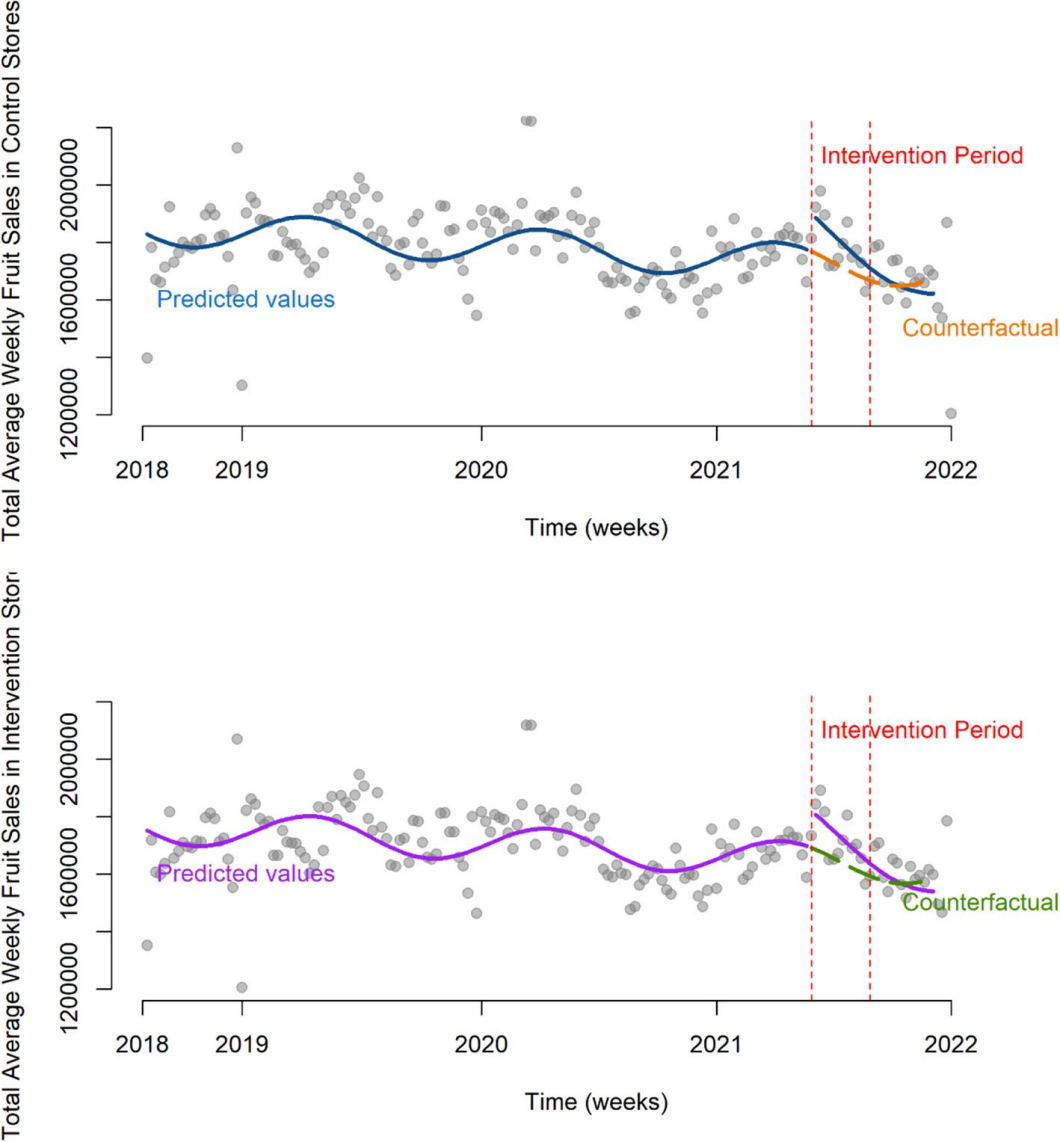
Mean sales of all fruit (in units) per week for control and intervention stores

Analyses of the change in ratio of all fruit sold in control versus intervention stores suggested the promotion of seasonal fruit did not have a significant increase on sales of the wider fruit category, and in fact the control stores had a slight increase in fruit purchased during this time comparative to the intervention stores (-0.01, 95% CI: -0.01–0.00, *p* = 0.02). There was no evidence of a change in general fruit unit sales between control and intervention stores (1.00, 95%CI: 0.97–1.03) (Fig. 5) (Supplementary Tables 9–10).

#### Seasonal fruit demographic analyses

**Fig. 6:**
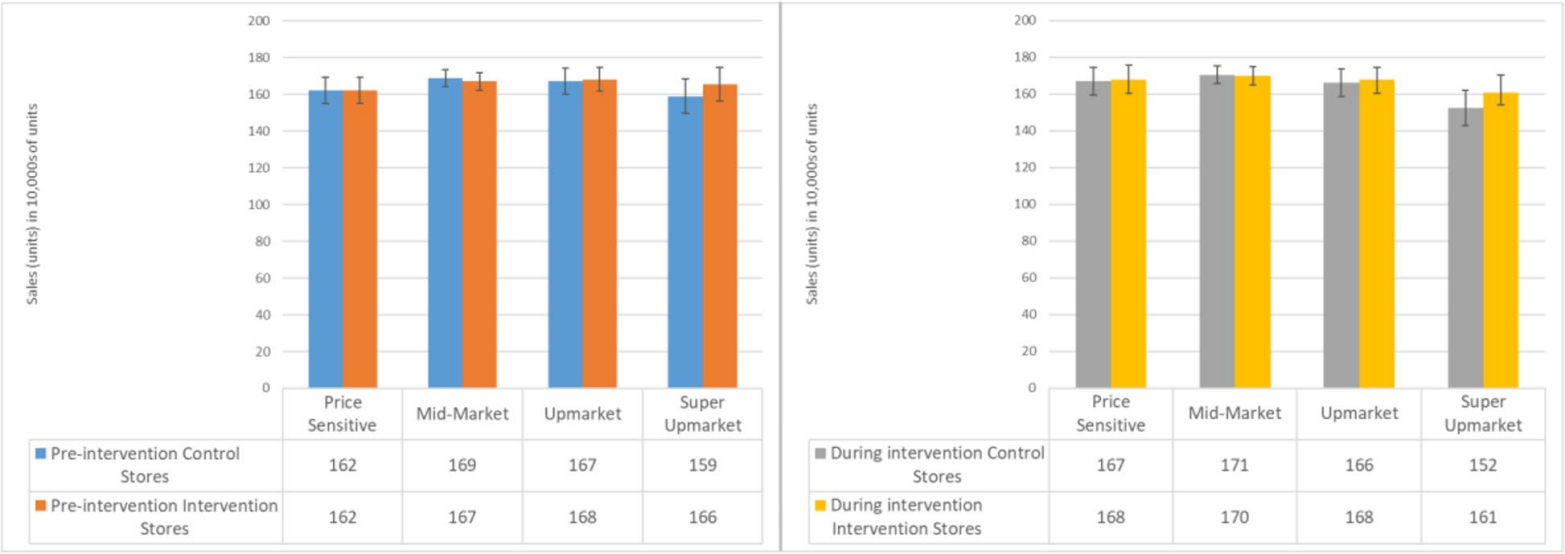
Marginal means of sales of fruit (units) before and during the intervention period in control and intervention stores

The intervention had a smaller impact on sales of promoted products in Super Upmarket stores (21% less, 95% CI: 9% to 31%) compared to the reference group of Mid-Market stores. Price sensitive stores did not significantly differ from Mid-Market (9% more, 95% CI: 2% less to 22% more). There were no significant interactions for the wider fruit category (Fig. 6) (Supplementary Table 10).

## Discussion

There was evidence that each set of promotional activity led to increases in the sales of promoted foods. However, there was little or no evidence of broader or sustained changes in purchasing patterns towards healthier or more sustainable products following these interventions. None of the promotional activities resulted in the continued purchase of promoted products after the intervention period was over, which suggest these interventions are limited in impact based on these short-term set ups.

The main strength of this study is that it is set in real-world grocery stores and reflects typical promotional activities. We were also able to access data on both promoted and related products to test for any compensatory effects. In-store supermarket research studies are rare due to the difficulty in implementation and data acquisition, and there is a lack of studies testing interventions to encourage both healthier and more sustainable diets. However, this context also brings some limitations. Some photos were provided of the promotional campaigns, but researchers had no control over the promotional activities implemented, were unable to conduct site visits due to the retrospective nature of the analysis, and were not made aware of the extent of any other forms of promotional activity on these goods before, or on any other goods during, the intervention time periods which could confound the analysis. For example, seasonal fruit often has a more prominent position in all stores during its period of availability and may have been promoted in previous years, which could dampen the impact of the promoted intervention. Researchers were also unaware of the positioning of other products in the store which may influence purchasing (e.g. if dairy milk and plant-based milk were next to each other or not). Socioeconomic position classification was provided with the data, but this was a store-level classification, not individual-level, which may not provide the granularity needed to assess SEP effects.

A further important point to note is that these interventions took place throughout 2021, when there were intermittent and sometimes prolonged lockdowns in the UK due to COVID-19 when hospitality was closed and purchasing patterns in grocery stores changed markedly. It was reported that as these restrictions eased, more people started buying products in smaller amounts, from more local stores, and possibly buying less due to a rebound in eating outside the home. These changes may explain the general decline in sales seen in some of the periods analysed, particularly for the Veganuary analysis [27, 28]. Visual inspection of the data hints at the impact of the COVID-19 pandemic. For example, for NAS plant-based milks there is a visible increase in purchasing of plant-based milks (often shelf-stable products) around the time of the spring 2020 lockdown when there was a fear of food shortages. There also appeared to be a general trend towards an increase in purchasing of plant-based milks and plant-based foods from 2018 to 2021 in control and intervention stores, independent of the promotional activity. In the absence of control stores we could not control for such effects.

This study supports previous research which suggests that although price promotions and monetary incentives are often found to be some of the more effective means of influencing food purchasing decisions [8], they have short-lived effects, increasing sales while they are applied but having inconsistent or unclear long-term impacts [18, 29, 30]. This is in contrast to more consistent evidence that removing promotions and making choice architecture changes to reduce the prominence on less healthy items, such as confectionery, have a sustained and positive impact [11, 13, 31]. Recent analysis on a Veganuary intervention much like the one analysed here showed a prolonged effect of the intervention on promoted items, however, the previous Veganuary intervention involved not only price promotions and prominent positioning, but also increased availability, with four new additional products. While prominent positioning and price promotions may not have been permanent, the introduction of new plant-based items that increases the availability and range of products seems likely to have been, and this may explain the extended intervention effect in the previous study [32]. Studies have consistently observed increased sales of promoted plant-based foods when promotions are in place, but no decreases in meat products which suggests increased purchases of promoted products rather than substitution for animal products, which may not achieve the intended shifts towards more sustainable diets [12, 32]. Further, promotions in this study focused on plant-based alternatives, such as plant-based meats, which are perceived to be relatively expensive [33] and there have been concerns expressed that they are highly processed with potentially higher levels of sodium than the meat options [33, 34]. Further research could consider how perceptions of promoted products may influence the impact of promotional activity.

Previous observational research has shown that when promotions are applied there is a greater increase in sales and purchasing of promoted products in affluent groups [25], but here the reverse effect was seen. Nakamura et al. analysed a wider range of promotions, including promotions implemented with even very small degrees of price reduction and showed that those with a higher SEP were more likely to increase purchasing of an item in response to promotional activity, hypothesising this may be due to a financial ability to stockpile or other non-monetary factors. The current study explored three relatively more substantive promotions and it is possible that the extent of promotional activity could contribute to differences in responsiveness. However, this needs further study. In the current study, purchases from the whole product category were analysed, as well as products that were directly promoted, and SEP measures were at the store rather than individual level which may also explain the differences in findings. Lower affluent stores typically sold less of these products generally, and the differential responses to the interventions were not sufficient to alter this overall pattern. This matches patterns found in another Veganuary analysis, where the intervention effect was greater in less affluent areas, though absolute sales remained lower than the most affluent areas [32]. As such, while the results of the current study suggest price promotions on healthier options may not exacerbate health inequalities, they are unlikely to mitigate against existing disparities.

In this study, analyses could only be conducted at a store-level and not in relation to individual purchaser characteristics. It is possible that differential effects, such as by age or income, could have resulted in a null overall effect and it highlights an area for future research. For example, these promotions might impact different age demographics in different ways. In the NAS plant-based milk intervention, the age classification of a store was consistently a significant factor in the association between the intervention and changes in purchasing patterns. Stores classified as young adult were observed to have a greater impact of the intervention and stores classified as retired having a smaller impact of the intervention compared to middle-aged stores. This could be due to various factors, such as product preference or disposable income, however, it could be an important consideration when applying such promotions. Further research at the individual level should consider if these patterns are occurring, or if there is a difference by age or by which products are promoted.

## Conclusion

Promotional activity (including prominent positioning and price promotions) on healthier or more sustainable food products can have a short-term impact on food purchases, but there is no evidence that intervention effects on behaviour are sustained.

### Supplementary Information

Supplementary Material 1.

Supplementary Material 2.

## Data Availability

This research was conducted according to a framework collaboration agreement between the University of Oxford and the food retailers. Access to the study dataset by external researchers is not permitted as this is defined as confidential information in the agreement. Access to the study data by external researchers will require the expressed written consent of the retailer  - please contact the corresponding author in the first instance.

## Abbreviations

NAS: No added sugars
SEP: Socioeconomic position
ITS: Interrupted time series
CGF: Consumer Goods Forum
IMD: Index of Multiple Deprivation
IRR: Incidence rate ratio
