# Supplementary material for: Do promotions of healthier or more sustainable foods increase sales? Findings from three natural experiments in UK supermarkets
Source: BMC Public Health. 2024 Jun 21;24:1658. doi: 10.1186/s12889-024-19080-x (PMC11191299; doi:10.1186/s12889-024-19080-x)
Supplement: Supplementary file 1 — Supplementary Material 1. [file 12889_2024_19080_MOESM1_ESM.docx]

Note: where there is an asterisk (*), this indicates this is the interaction term of the two variables listed.

Supplementary Table 1. Interrupted time series models for plant-based milk intervention.

| **Model** | **Terms** | **Coefficient** | **Standard Error** | **p-value** | **Lower 95%** | **Upper 95%** |
| --- | --- | --- | --- | --- | --- | --- |
| Sales (£) of promoted plant-based milk | Constant | 60.776 | 6.673 | <0.001 | 47.587 | 73.966 |
|  | Time (weeks) | 0.323 | 0.086 | <0.001 | 0.154 | 0.493 |
|  | Intervention | 103.225 | 8.236 | <0.001 | 86.947 | 119.504 |
|  | Time (weeks) * intervention | -25.318 | 1.648 | <0.001 | -28.575 | -22.061 |
| Sales (units) of promoted plant-based milk | Constant | 50.854 | 8.475 | <0.001 | 34.104 | 67.605 |
|  | Time (weeks) | 0.211 | 0.113 | 0.064 | -0.012 | 0.434 |
|  | Intervention | 126.094 | 10.863 | <0.001 | 104.622 | 147.566 |
|  | Time (weeks) * intervention | -26.562 | 2.511 | <0.001 | -31.525 | -21.599 |
| Sales (£) of all no added sugar plant-based milk | Constant | 518.523 | 9.883 | <0.001 | 498.989 | 538.057 |
|  | Time (weeks) | 1.678 | 0.137 | <0.001 | 1.407 | 1.949 |
|  | Intervention | 265.392 | 16.791 | <0.001 | 232.204 | 298.581 |
|  | Time (weeks) * intervention | -85.504 | 1.747 | <0.001 | -88.957 | -82.050 |
| Sales (units) of all no added sugar plant-based milk | Constant | 443.324 | 14.365 | <0.001 | 414.930 | 471.719 |
|  | Time (weeks) | 1.340 | 0.199 | <0.001 | 0.946 | 1.734 |
|  | Intervention | 306.544 | 21.362 | <0.001 | 264.320 | 348.767 |
|  | Time (weeks) * intervention | -79.974 | 3.352 | <0.001 | -86.599 | -73.349 |
| Sales (£) of all plant-based milk | Constant | 1523.551 | 28.707 | <0.001 | 1466.810 | 1580.291 |
|  | Time (weeks) | 4.055 | 0.403 | <0.001 | 3.258 | 4.853 |
|  | Intervention | 398.707 | 56.396 | <0.001 | 287.236 | 510.179 |
|  | Time (weeks) * intervention | -136.678 | 1.811 | <0.001 | -140.256 | -133.099 |
| Sales (units) of all plant-based milk | Constant | 1206.454 | 30.955 | <0.001 | 1145.269 | 1267.639 |
|  | Time (weeks) | 2.763 | 0.471 | <0.001 | 1.832 | 3.694 |
|  | Intervention | 462.741 | 55.436 | <0.001 | 353.167 | 572.315 |
|  | Time (weeks) * intervention | -121.419 | 5.702 | <0.001 | -132.688 | -110.149 |

Supplementary Table 2. Multivariable hierarchical mixed-effects models for plant-based milk intervention.

| **Model** |  | **Term** | **Coefficient** | **Standard Error** | **p-value** | **Lower 95%** | **Upper 95%** |
| --- | --- | --- | --- | --- | --- | --- | --- |
| Sales of promoted plant-based milks (£) (with interaction terms) |  | Constant | 52.71 | 4.44 | <0.001 | 43.95 | 61.46 |
|  |  | Time (weeks) | 0.33 | 0.01 | <0.001 | 0.32 | 0.35 |
|  |  | Intervention Period | 37.92 | 2.35 | <0.001 | 33.32 | 42.52 |
|  | Retailer Affluence Group (Reference: Mid-Market) | Price Sensitive | -16.20 | 6.73 | 0.017 | -29.45 | -2.95 |
|  |  | Super Upmarket | 18.76 | 10.60 | 0.078 | -2.12 | 39.64 |
|  |  | Upmarket | 4.81 | 8.78 | 0.585 | -12.49 | 22.11 |
|  | Retailer Age Groups (Reference: Middle Aged) | Families | 26.97 | 8.19 | 0.001 | 10.83 | 43.10 |
|  |  | Retired | -35.30 | 17.26 | 0.042 | -69.29 | -1.31 |
|  |  | Young Adults | 23.37 | 6.21 | <0.001 | 11.14 | 35.61 |
|  |  | Intervention * Price Sensitive | -1.94 | 4.37 | 0.657 | -10.51 | 6.63 |
|  |  | Intervention * Super Upmarket | 6.83 | 6.71 | 0.309 | -6.33 | 19.98 |
|  |  | Intervention * Upmarket | -0.38 | 5.69 | 0.946 | -11.54 | 10.77 |
|  |  |  |  |  |  |  |  |
| Sales of NAS plant-based milks (£) (with interaction terms) |  | Constant | 531.19 | 31.38 | <0.001 | 469.38 | 593.00 |
|  |  | Time (weeks) | 1.58 | 0.02 | <0.001 | 1.54 | 1.61 |
|  |  | Intervention Period | 64.65 | 5.46 | <0.001 | 53.94 | 75.35 |
|  | Retailer Affluence Group (Reference: Mid-Market) | Price Sensitive | -222.63 | 47.79 | <0.001 | -316.76 | -128.50 |
|  |  | Super Upmarket | 165.88 | 75.29 | 0.029 | 17.57 | 314.18 |
|  |  | Upmarket | 53.91 | 62.39 | 0.389 | -68.98 | 176.80 |
|  | Retailer Age Groups (Reference: Middle Aged) | Families | 114.67 | 58.19 | 0.050 | 0.05 | 229.28 |
|  |  | Retired | -275.14 | 122.58 | 0.026 | -516.58 | -33.70 |
|  |  | Young Adults | 120.57 | 44.12 | 0.007 | 33.67 | 207.48 |
|  |  | Intervention * Price Sensitive | -22.10 | 10.18 | 0.030 | -42.06 | -2.15 |
|  |  | Intervention * Super Upmarket | -63.87 | 15.63 | <0.001 | -94.50 | -33.24 |
|  |  | Intervention * Upmarket | -40.05 | 13.25 | 0.003 | -66.01 | -14.08 |
| Sales of all plant-based milks (£) (with interaction terms) |  | Constant | 1460.04 | 100.35 | <0.001 | 1262.38 | 1657.70 |
|  |  | Time (weeks) | 4.15 | 0.04 | <0.001 | 4.06 | 4.23 |
|  |  | Intervention Period | 74.91 | 14.90 | <0.001 | 45.69 | 104.12 |
|  | Retailer Affluence Group (Reference: Mid-Market) | Price Sensitive | -588.81 | 152.85 | <0.001 | -889.87 | -287.75 |
|  |  | Super Upmarket | 422.90 | 240.81 | 0.081 | -51.44 | 897.23 |
|  |  | Upmarket | 57.31 | 199.54 | 0.774 | -335.72 | 450.34 |
|  | Retailer Age Groups (Reference: Middle Aged) | Families | 398.07 | 186.11 | 0.034 | 31.49 | 764.65 |
|  |  | Retired | -829.04 | 392.04 | 0.036 | -1601.25 | -56.84 |
|  |  | Young Adults | 465.84 | 141.12 | 0.001 | 187.88 | 743.80 |
|  |  | Intervention * Price Sensitive | -78.23 | 27.79 | 0.005 | -132.70 | -23.77 |
|  |  | Intervention * Super Upmarket | 35.77 | 42.65 | 0.402 | -47.83 | 119.36 |
|  |  | Intervention * Upmarket | -43.53 | 36.16 | 0.229 | -114.40 | 27.34 |

Supplementary Table 3. Multivariate hierarchical mixed-effects models for plant-based milk interventions (negative binomial models).

| **Model** |  | **Term** | **Coefficient** | **Standard Error** | **p-value** | **Lower 95%** | **Upper 95%** |
| --- | --- | --- | --- | --- | --- | --- | --- |
| Sales of promoted plant-based milks (units) (with interaction terms) |  | Constant | 3.75 | 0.05 | <0.001 | 3.66 | 3.85 |
|  |  | Time (weeks) | 0.00 | 0.00 | <0.001 | 0.00 | 0.00 |
|  |  | Intervention Period | 0.47 | 0.03 | <0.001 | 0.41 | 0.52 |
|  | Retailer Affluence Group (Reference: Mid-Market) | Price Sensitive | -0.21 | 0.07 | 0.004 | -0.35 | -0.07 |
|  |  | Super Upmarket | 0.21 | 0.11 | 0.066 | -0.01 | 0.43 |
|  |  | Upmarket | 0.07 | 0.09 | 0.457 | -0.11 | 0.26 |
|  | Retailer Age Groups (Reference: Middle Aged) | Families | 0.31 | 0.09 | <0.001 | 0.14 | 0.48 |
|  |  | Retired | -0.57 | 0.19 | 0.002 | -0.93 | -0.20 |
|  |  | Young Adults | 0.25 | 0.07 | <0.001 | 0.12 | 0.38 |
|  |  | Intervention * Price Sensitive | 0.07 | 0.05 | 0.186 | -0.03 | 0.17 |
|  |  | Intervention * Super Upmarket | -0.04 | 0.08 | 0.612 | -0.20 | 0.12 |
|  |  | Intervention * Upmarket | 0.01 | 0.07 | 0.899 | -0.12 | 0.14 |
| Sales of NAS plant-based milks (units) (with interaction terms) |  | Constant | 6.05 | 0.05 | <0.001 | 5.95 | 6.14 |
|  |  | Time (weeks) | 0.00 | 0.00 | <0.001 | 0.00 | 0.00 |
|  |  | Intervention Period | 0.16 | 0.01 | <0.001 | 0.14 | 0.17 |
|  | Retailer Affluence Group (Reference: Mid-Market) | Price Sensitive | -0.38 | 0.07 | <0.001 | -0.52 | -0.24 |
|  |  | Super Upmarket | 0.19 | 0.11 | 0.096 | -0.03 | 0.41 |
|  |  | Upmarket | 0.08 | 0.09 | 0.391 | -0.10 | 0.27 |
|  | Retailer Age Groups (Reference: Middle Aged) | Families | 0.15 | 0.09 | 0.086 | -0.02 | 0.32 |
|  |  | Retired | -0.53 | 0.19 | 0.004 | -0.90 | -0.17 |
|  |  | Young Adults | 0.18 | 0.07 | 0.006 | 0.05 | 0.31 |
|  |  | Intervention * Price Sensitive | 0.07 | 0.02 | <0.001 | 0.04 | 0.10 |
|  |  | Intervention * Super Upmarket | -0.11 | 0.02 | <0.001 | -0.16 | -0.06 |
|  |  | Intervention * Upmarket | -0.07 | 0.02 | 0.001 | -0.11 | -0.03 |
| Sales of all plant-based milks (units) (with interaction terms) |  | Constant | 6.96 | 0.05 | 0.000 | 6.86 | 7.07 |
|  |  | Time (weeks) | 0.00 | 0.00 | 0.000 | 0.00 | 0.00 |
|  |  | Intervention Period | 0.09 | 0.01 | 0.000 | 0.07 | 0.10 |
|  | Retailer Affluence Group (Reference: Mid-Market) | Price Sensitive | -0.36 | 0.08 | 0.000 | -0.51 | -0.20 |
|  |  | Super Upmarket | 0.16 | 0.12 | 0.188 | -0.08 | 0.41 |
|  |  | Upmarket | 0.05 | 0.10 | 0.632 | -0.15 | 0.25 |
|  | Retailer Age Groups (Reference: Middle Aged) | Families | 0.22 | 0.10 | 0.020 | 0.03 | 0.41 |
|  |  | Retired | -0.58 | 0.20 | 0.004 | -0.98 | -0.19 |
|  |  | Young Adults | 0.24 | 0.07 | 0.001 | 0.10 | 0.38 |
|  |  | Intervention * Price Sensitive | 0.04 | 0.01 | 0.006 | 0.01 | 0.07 |
|  |  | Intervention * Super Upmarket | -0.03 | 0.02 | 0.142 | -0.07 | 0.01 |
|  |  | Intervention * Upmarket | -0.04 | 0.02 | 0.041 | -0.07 | 0.00 |

Supplementary Table 4. Multivariate hierarchical mixed-effects models for plant-based milk intervention using index of multiple deprivations (IMD) for affluence.

| **Model** |  | **Term** | **Coefficient** | **Standard Error** | **p-value** | **Lower 95%** | **Upper 95%** |
| --- | --- | --- | --- | --- | --- | --- | --- |
| Sales of promoted plant-based milks (£) (with IMD interaction terms) |  | Constant | 64.98 | 4.98 | <0.001 | 55.18 | 74.78 |
|  |  | Time (weeks) | 0.33 | 0.01 | <0.001 | 0.32 | 0.35 |
|  |  | Intervention | 41.44 | 3.05 | <0.001 | 35.45 | 47.42 |
|  | Index of Multiple Deprivation Group (ref = Mid) | High | -15.90 | 6.98 | 0.024 | -29.64 | -2.16 |
|  |  | Low | -27.43 | 6.17 | <0.001 | -39.58 | -15.27 |
|  | Retailer Age Groups (Reference: Middle Aged) | Families | 33.76 | 7.95 | <0.001 | 18.09 | 49.42 |
|  |  | Retired | -36.67 | 16.83 | 0.031 | -69.82 | -3.53 |
|  |  | Young Adults | 24.76 | 6.05 | <0.001 | 12.84 | 36.69 |
|  |  | Intervention*IMD (High) | -9.83 | 4.61 | 0.033 | -18.86 | -0.80 |
|  |  | Intervention* IMD (Low) | -2.40 | 4.08 | 0.556 | -10.39 | 5.59 |
| Sales of promoted plant-based milks (units) (with IMD interaction terms) |  | Constant | 3.87 | 0.05 | <0.001 | 3.76 | 3.97 |
|  |  | Time (weeks) | 0.00 | 0.00 | <0.001 | 0.00 | 0.00 |
|  |  | Intervention | 0.46 | 0.04 | <0.001 | 0.39 | 0.54 |
|  | Index of Multiple Deprivation Group (ref = Mid) | High | -0.13 | 0.08 | 0.094 | -0.28 | 0.02 |
|  |  | Low | -0.29 | 0.07 | <0.001 | -0.42 | -0.16 |
|  | Retailer Age Groups (Reference: Middle Aged) | Families | 0.38 | 0.09 | <0.001 | 0.21 | 0.55 |
|  |  | Retired | -0.57 | 0.18 | 0.002 | -0.92 | -0.21 |
|  |  | Young Adults | 0.27 | 0.07 | <0.001 | 0.14 | 0.39 |
|  |  | Intervention*IMD (High) | -0.04 | 0.05 | 0.478 | -0.15 | 0.07 |
|  |  | Intervention* IMD (Low) | 0.07 | 0.05 | 0.164 | -0.03 | 0.16 |
| Sales of NAS plant-based milks (£) (with IMD interaction terms) |  | Constant | 561.96 | 35.49 | <0.001 | 492.07 | 631.84 |
|  |  | Time (weeks) | 1.68 | 0.02 | <0.001 | 1.65 | 1.71 |
|  |  | Intervention | 59.30 | 6.76 | <0.001 | 46.04 | 72.56 |
|  | Index of Multiple Deprivation Group (ref = Mid) | High | -45.20 | 50.59 | 0.373 | -144.83 | 54.43 |
|  |  | Low | -215.82 | 44.32 | <0.001 | -303.12 | -128.53 |
|  | Retailer Age Groups (Reference: Middle Aged) | Families | 174.85 | 58.01 | 0.003 | 60.60 | 289.10 |
|  |  | Retired | -233.41 | 123.06 | 0.059 | -475.77 | 8.95 |
|  |  | Young Adults | 129.72 | 43.74 | 0.003 | 43.57 | 215.87 |
|  |  | Intervention*IMD (High) | -27.12 | 10.34 | 0.009 | -47.39 | -6.84 |
|  |  | Intervention* IMD (Low) | -2.12 | 9.07 | 0.815 | -19.90 | 15.66 |
| Sales of all plant-based milks (£) (with IMD interaction terms) |  | Constant | 1624.55 | 113.77 | <0.001 | 1400.48 | 1848.61 |
|  |  | Time (weeks) | 4.05 | 0.04 | <0.001 | 3.97 | 4.14 |
|  |  | Intervention | 69.11 | 18.88 | <0.001 | 32.10 | 106.11 |
|  | Index of Multiple Deprivation Group (ref = Mid) | High | -256.39 | 162.21 | 0.116 | -575.85 | 63.07 |
|  |  | Low | -595.70 | 142.12 | <0.001 | -875.61 | -315.79 |
|  | Retailer Age Groups (Reference: Middle Aged) | Families | 572.95 | 186.01 | 0.002 | 206.60 | 939.30 |
|  |  | Retired | -747.95 | 394.59 | 0.059 | -1525.08 | 29.18 |
|  |  | Young Adults | 500.45 | 140.26 | 0.000 | 224.21 | 776.69 |
|  |  | Intervention*IMD (High) | -15.88 | 28.87 | 0.582 | -72.47 | 40.71 |
|  |  | Intervention* IMD (Low) | -19.61 | 25.32 | 0.439 | -69.24 | 30.02 |
| Sales of NAS plant-based milks (units) (with IMD interaction terms) |  | Constant | 6.05 | 0.06 | <0.001 | 5.94 | 6.16 |
|  |  | Time (weeks) | 0.00 | 0.00 | <0.001 | 0.00 | 0.00 |
|  |  | Intervention | 0.14 | 0.01 | <0.001 | 0.12 | 0.16 |
|  | Index of Multiple Deprivation Group (ref = Mid) | High | -0.04 | 0.08 | 0.640 | -0.20 | 0.12 |
|  |  | Low | -0.32 | 0.07 | <0.001 | -0.46 | -0.18 |
|  | Retailer Age Groups (Reference: Middle Aged) | Families | 0.25 | 0.09 | 0.009 | 0.06 | 0.43 |
|  |  | Retired | -0.44 | 0.20 | 0.026 | -0.83 | -0.05 |
|  |  | Young Adults | 0.21 | 0.07 | 0.003 | 0.07 | 0.35 |
|  |  | Intervention*IMD (High) | -0.04 | 0.02 | 0.018 | -0.07 | -0.01 |
|  |  | Intervention* IMD (Low) | 0.07 | 0.01 | <0.001 | 0.04 | 0.09 |
| Sales of all plant-based milks (units) (with IMD interaction terms) |  | Constant | 7.01 | 0.06 | <0.001 | 6.89 | 7.13 |
|  |  | Time (weeks) | 0.00 | 0.00 | <0.001 | 0.00 | 0.00 |
|  |  | Intervention | 0.08 | 0.01 | <0.001 | 0.06 | 0.10 |
|  | Index of Multiple Deprivation Group (ref = Mid) | High | -0.08 | 0.09 | 0.374 | -0.25 | 0.09 |
|  |  | Low | -0.30 | 0.08 | <0.001 | -0.45 | -0.15 |
|  | Retailer Age Groups (Reference: Middle Aged) | Families | 0.31 | 0.10 | 0.002 | 0.12 | 0.51 |
|  |  | Retired | -0.51 | 0.21 | 0.017 | -0.92 | -0.09 |
|  |  | Young Adults | 0.27 | 0.08 | <0.001 | 0.12 | 0.41 |
|  |  | Intervention*IMD (High) | -0.01 | 0.02 | 0.444 | -0.04 | 0.02 |
|  |  | Intervention* IMD (Low) | 0.05 | 0.01 | <0.001 | 0.02 | 0.07 |

Supplementary Table 5. Interrupted time series analysis for Veganuary promoted products

| **Model** | **Term** | **Coefficient** | **Standard Error** | **p-value** | **Lower 95%** | **Upper 95%** |
| --- | --- | --- | --- | --- | --- | --- |
| Sales (units) of promoted Veganuary products | Constant | 14.786 | 8.942 | 0.100 | -2.845 | 32.416 |
|  | Time (weeks) | 0.947 | 0.095 | <0.001 | 0.760 | 1.134 |
|  | Intervention | 60.483 | 11.832 | <0.001 | 37.154 | 83.812 |
|  | Time*intervention | -3.202 | 0.335 | <0.001 | -3.863 | -2.542 |
|  | Month (January) | 37.439 | 14.559 | 0.011 | 8.732 | 66.145 |
|  | Last week of the year | -38.574 | 5.855 | <0.001 | -50.117 | -27.030 |
| Sales (£) of promoted Veganuary products | Constant | 1.633 | 23.269 | 0.944 | -44.246 | 47.513 |
|  | Time (weeks) | 2.071 | 0.239 | <0.001 | 1.599 | 2.542 |
|  | Intervention | 148.411 | 30.286 | <0.001 | 88.695 | 208.128 |
|  | Time*intervention | -7.017 | 0.694 | <0.001 | -8.384 | -5.649 |
|  | Month (January) | 58.684 | 30.663 | 0.057 | -1.774 | 119.142 |
|  | Last week of the year | -67.625 | 13.872 | <0.001 | -94.976 | -40.274 |

Supplementary Table 6. Interrupted time series analyses for specific food categories during the Veganuary intervention.

| **Model** | **Term** | **Coefficient** | **Standard Error** | **p-value** | **Lower**  **95%** | **Upper**  **95%** |
| --- | --- | --- | --- | --- | --- | --- |
| Sales (units) of dairy products | Constant | 1241776.916 | 17294.906 | <0.001 | 1207676.224 | 1275877.608 |
|  | Time (weeks) | -926.118 | 208.077 | <0.001 | -1336.388 | -515.849 |
|  | Intervention | 93342.254 | 33163.651 | 0.005 | 27952.858 | 158731.649 |
|  | Time*intervention | -3312.595 | 676.208 | <0.001 | -4645.886 | -1979.304 |
|  | Month (January) | -95062.756 | 16174.488 | <0.001 | -126954.299 | -63171.214 |
|  | Last week of the year | -176524.593 | 109223.702 | 0.108 | -391883.025 | 38833.839 |
| Sales (£) of dairy products | Constant | 1819212.235 | 33398.494 | <0.001 | 1753359.795 | 1885064.675 |
|  | Time (weeks) | -584.409 | 447.518 | 0.193 | -1466.789 | 297.972 |
|  | Intervention | 224448.345 | 63742.055 | 0.001 | 98766.933 | 350129.757 |
|  | Time*intervention | -4772.784 | 1482.888 | 0.001 | -7696.622 | -1848.946 |
|  | Month (January) | -167156.599 | 27091.851 | <0.001 | -220574.112 | -113739.086 |
|  | Last week of the year | -176470.611 | 182972.855 | 0.336 | -537241.638 | 184300.415 |
| Sales (units) of plant-based dairy products | Constant | 19519.659 | 2101.976 | <0.001 | 15375.154 | 23664.164 |
|  | Time (weeks) | 344.802 | 16.846 | <0.001 | 311.587 | 378.016 |
|  | Intervention | -1130.903 | 2378.593 | 0.635 | -5820.820 | 3559.013 |
|  | Time*intervention | -887.284 | 56.178 | <0.001 | -998.051 | -776.517 |
|  | Month (January) | -1383.779 | 2840.741 | 0.627 | -6984.923 | 4217.364 |
|  | Last week of the year | -1751.685 | 4295.105 | 0.684 | -10220.424 | 6717.053 |
| Sales (£) of plant-based dairy products | Constant | 22226.845 | 3724.073 | <0.001 | 14884.020 | 29569.669 |
|  | Time (weeks) | 498.430 | 25.066 | <0.001 | 449.006 | 547.854 |
|  | Intervention | 3485.275 | 3082.789 | 0.260 | -2593.119 | 9563.668 |
|  | Time*intervention | -1197.758 | 71.314 | <0.001 | -1338.369 | -1057.147 |
|  | Month (January) | -3675.497 | 4005.170 | 0.360 | -11572.565 | 4221.572 |
|  | Last week of the year | -8739.692 | 4331.877 | 0.045 | -17280.935 | -198.448 |
| Sales (£) of meat products | Constant | 5283003.082 | 165034.256 | <0.001 | 4957601.928 | 5608404.235 |
|  | Time (weeks) | -50.143 | 1686.362 | 0.976 | -3375.174 | 3274.888 |
|  | Intervention | -150988.051 | 217735.298 | 0.489 | -580300.842 | 278324.741 |
|  | Time*intervention | -22317.170 | 5848.831 | <0.001 | -33849.420 | -10784.920 |
|  | Month (January) | -316820.530 | 318626.770 | 0.321 | -945062.938 | 311421.879 |
|  | Last week of the year | -619841.977 | 184871.565 | 0.001 | -984356.727 | -255327.228 |
| Sales (£) of meat alternative products | Constant | 57191.054 | 1107.753 | <0.001 | 55006.876 | 59375.232 |
|  | Time (weeks) | 78.236 | 17.702 | <0.001 | 43.332 | 113.140 |
|  | Intervention | 1234.766 | 2502.372 | 0.622 | -3699.207 | 6168.740 |
|  | Time*intervention | -242.729 | 42.276 | <0.001 | -326.084 | -159.373 |
|  | Month (January) | 1845.168 | 2341.244 | 0.432 | -2771.108 | 6461.444 |
|  | Last week of the year | -14317.110 | 2621.473 | <0.001 | -19485.918 | -9148.302 |
| Sales (units) of meat products | Constant | 1971672.667 | 54043.499 | <0.001 | 1865114.081 | 2078231.254 |
|  | Time (weeks) | -34.655 | 591.294 | 0.953 | -1200.521 | 1131.211 |
|  | Intervention | 65704.004 | 81129.529 | 0.419 | -94260.618 | 225668.626 |
|  | Time*intervention | -9009.059 | 2025.626 | <0.001 | -13003.023 | -5015.094 |
|  | Month (January) | -149728.301 | 110255.664 | 0.176 | -367121.471 | 67664.869 |
|  | Last week of the year | -210996.576 | 96483.944 | 0.030 | -401235.787 | -20757.365 |
| Sales (units) of meat alternative products | Constant | 28708.123 | 484.290 | <0.001 | 27753.238 | 29663.008 |
|  | Time (weeks) | 17.808 | 7.367 | 0.017 | 3.283 | 32.334 |
|  | Intervention | 1402.510 | 1091.200 | 0.200 | -749.029 | 3554.050 |
|  | Time*intervention | -114.442 | 20.616 | <0.001 | -155.091 | -73.792 |
|  | Month (January) | 1145.015 | 1219.090 | 0.349 | -1258.688 | 3548.718 |
|  | Last week of the year | -6830.193 | 1617.769 | <0.001 | -10019.979 | -3640.407 |

Supplementary Table 7. Multivariate hierarchical mixed-effects models for Veganuary (sales in £).

| **Model** |  | **Term** | **Coefficient** | **Standard Error** | **p-value** | **Lower**  **95%** | **Upper**  **95%** |
| --- | --- | --- | --- | --- | --- | --- | --- |
| Sales of promoted products (£) in Veganuary (with interaction terms) |  | Constant | 10.08 | 8.96 | 0.263 | -7.66 | 27.82 |
|  |  | Time (weeks) | 2.00 | 0.01 | <0.001 | 1.98 | 2.02 |
|  |  | Intervention Period | 263.48 | 4.20 | <0.001 | 255.24 | 271.71 |
|  | Retailer Affluence Group (Reference: Mid-Market) | Price Sensitive | -49.87 | 15.55 | 0.002 | -80.65 | -19.09 |
|  |  | Super Upmarket | 72.30 | 23.96 | 0.003 | 24.87 | 119.73 |
|  |  | Upmarket | 43.77 | 18.91 | 0.023 | 6.33 | 81.20 |
|  | Retailer Age Groups (Reference: Middle Aged) | Families | -12.16 | 16.73 | 0.469 | -45.27 | 20.96 |
|  |  | Retired | -38.41 | 39.71 | 0.336 | -117.03 | 40.21 |
|  |  | Young Adults | 6.68 | 12.99 | 0.609 | -19.05 | 32.40 |
|  |  | Intervention * Price Sensitive | -78.08 | 9.16 | <0.001 | -96.03 | -60.13 |
|  |  | Intervention * Super Upmarket | 160.30 | 13.99 | <0.001 | 132.88 | 187.71 |
|  |  | Intervention * Upmarket | 84.04 | 11.14 | <0.001 | 62.20 | 105.87 |
| Sales of meat alternatives (£) in Veganuary (with interaction terms) |  | Constant | 633.70 | 33.97 | <0.001 | 566.45 | 700.94 |
|  |  | Time (weeks) | 0.76 | 0.02 | <0.001 | 0.72 | 0.80 |
|  |  | Intervention Period | -10.98 | 6.90 | 0.112 | -24.51 | 2.55 |
|  | Retailer Affluence Group (Reference: Mid-Market) | Price Sensitive | -176.18 | 59.18 | 0.004 | -293.34 | -59.01 |
|  |  | Super Upmarket | 91.98 | 91.21 | 0.316 | -88.59 | 272.54 |
|  |  | Upmarket | 42.22 | 71.99 | 0.559 | -100.30 | 184.74 |
|  | Retailer Age Groups (Reference: Middle Aged) | Families | -69.90 | 63.69 | 0.275 | -195.98 | 56.18 |
|  |  | Retired | -200.08 | 151.18 | 0.189 | -499.39 | 99.22 |
|  |  | Young Adults | -5.29 | 49.47 | 0.915 | -103.23 | 92.66 |
|  |  | Intervention * Price Sensitive | -4.16 | 15.04 | 0.782 | -33.65 | 25.32 |
|  |  | Intervention * Super Upmarket | 116.22 | 22.98 | <0.001 | 71.19 | 161.26 |
|  |  | Intervention * Upmarket | 79.80 | 18.30 | <0.001 | 43.93 | 115.68 |
| Sales of meat (£) in Veganuary (with interaction terms) |  | Constant | 56575.69 | 2827.83 | <0.001 | 50977.61 | 62173.76 |
|  |  | Time (weeks) | 0.98 | 1.83 | 0.591 | -2.60 | 4.57 |
|  |  | Intervention Period | -3723.33 | 654.29 | <0.001 | -5005.79 | -2440.86 |
|  | Retailer Affluence Group (Reference: Mid-Market) | Price Sensitive | -10291.87 | 4925.32 | 0.039 | -20042.69 | -541.05 |
|  |  | Super Upmarket | 7906.01 | 7590.72 | 0.300 | -7121.58 | 22933.61 |
|  |  | Upmarket | 4366.80 | 5991.16 | 0.468 | -7494.10 | 16227.69 |
|  | Retailer Age Groups (Reference: Middle Aged) | Families | -1205.30 | 5300.17 | 0.821 | -11698.24 | 9287.64 |
|  |  | Retired | -15939.35 | 12582.12 | 0.208 | -40848.62 | 8969.93 |
|  |  | Young Adults | -2537.36 | 4117.34 | 0.539 | -10688.60 | 5613.88 |
|  |  | Intervention * Price Sensitive | 2334.69 | 1426.02 | 0.102 | -460.43 | 5129.81 |
|  |  | Intervention * Super Upmarket | -2445.04 | 2178.28 | 0.262 | -6714.66 | 1824.57 |
|  |  | Intervention * Upmarket | -1140.77 | 1734.82 | 0.511 | -4541.17 | 2259.64 |
| Sales of plant-based dairy (£) in Veganuary (with interaction terms) |  | Constant | 235.19 | 34.24 | <0.001 | 167.41 | 302.97 |
|  |  | Time (weeks) | 5.21 | 0.03 | <0.001 | 5.15 | 5.26 |
|  |  | Intervention Period | -22.00 | 10.34 | 0.033 | -42.27 | -1.74 |
|  | Retailer Affluence Group (Reference: Mid-Market) | Price Sensitive | -216.45 | 59.58 | <0.001 | -334.40 | -98.49 |
|  |  | Super Upmarket | 224.14 | 91.82 | 0.016 | 42.35 | 405.93 |
|  |  | Upmarket | 105.80 | 72.47 | 0.148 | -37.68 | 249.28 |
|  | Retailer Age Groups (Reference: Middle Aged) | Families | -8.26 | 64.11 | 0.898 | -135.19 | 118.67 |
|  |  | Retired | -126.99 | 152.20 | 0.406 | -428.31 | 174.34 |
|  |  | Young Adults | 18.24 | 49.81 | 0.715 | -80.37 | 116.84 |
|  |  | Intervention * Price Sensitive | -95.03 | 22.53 | <0.001 | -139.20 | -50.87 |
|  |  | Intervention * Super Upmarket | 171.82 | 34.42 | <0.001 | 104.36 | 239.28 |
|  |  | Intervention * Upmarket | 82.09 | 27.41 | 0.003 | 28.36 | 135.81 |
| Sales of dairy (£) in Veganuary (with interaction terms) |  | Constant | 19188.71 | 904.20 | <0.001 | 17398.67 | 20978.75 |
|  |  | Time (weeks) | -4.93 | 0.35 | <0.001 | -5.62 | -4.24 |
|  |  | Intervention Period | 199.16 | 126.14 | 0.114 | -48.07 | 446.40 |
|  | Retailer Affluence Group (Reference: Mid-Market) | Price Sensitive | -4486.22 | 1576.15 | 0.005 | -7606.58 | -1365.86 |
|  |  | Super Upmarket | 5031.03 | 2429.11 | 0.041 | 222.05 | 9840.02 |
|  |  | Upmarket | 1728.82 | 1917.23 | 0.369 | -2066.78 | 5524.43 |
|  | Retailer Age Groups (Reference: Middle Aged) | Families | -681.46 | 1696.14 | 0.689 | -4039.36 | 2676.44 |
|  |  | Retired | -3842.15 | 4026.47 | 0.342 | -11813.50 | 4129.20 |
|  |  | Young Adults | -217.99 | 1317.61 | 0.869 | -2826.51 | 2390.53 |
|  |  | Intervention * Price Sensitive | 666.04 | 274.91 | 0.015 | 127.20 | 1204.89 |
|  |  | Intervention * Super Upmarket | 62.53 | 419.93 | 0.882 | -760.58 | 885.63 |
|  |  | Intervention * Upmarket | 141.10 | 334.44 | 0.673 | -514.44 | 796.63 |

Supplementary Table 8. Multivariate hierarchical mixed-effects models for Veganuary (sales in units).

| **Model** |  | **Term** | **Coefficient** | **Standard Error** | **p-value** | **Lower**  **95%** | **Upper**  **95%** |
| --- | --- | --- | --- | --- | --- | --- | --- |
| Sales of promoted products (units) in Veganuary (with interaction terms) |  | Constant | 3.57 | 0.05 | <0.001 | 3.47 | 3.67 |
|  |  | Time (weeks) | 0.01 | 0.00 | <0.001 | 0.01 | 0.01 |
|  |  | Intervention Period | 0.44 | 0.02 | <0.001 | 0.41 | 0.48 |
|  | Retailer Affluence Group (Reference: Mid-Market) | Price Sensitive | -0.37 | 0.09 | <0.001 | -0.54 | -0.20 |
|  |  | Super Upmarket | 0.41 | 0.13 | 0.002 | 0.15 | 0.68 |
|  |  | Upmarket | 0.24 | 0.11 | 0.021 | 0.04 | 0.45 |
|  | Retailer Age Groups (Reference: Middle Aged) | Families | -0.09 | 0.09 | 0.357 | -0.27 | 0.10 |
|  |  | Retired | -0.22 | 0.22 | 0.326 | -0.65 | 0.22 |
|  |  | Young Adults | 0.05 | 0.07 | 0.521 | -0.10 | 0.19 |
|  |  | Intervention * Price Sensitive | 0.14 | 0.04 | <0.001 | 0.07 | 0.22 |
|  |  | Intervention * Super Upmarket | -0.07 | 0.06 | 0.212 | -0.18 | 0.04 |
|  |  | Intervention * Upmarket | -0.05 | 0.05 | 0.226 | -0.14 | 0.03 |
| Sales of meat alternatives (units) in Veganuary (with interaction terms) |  | Constant | 5.71 | 0.05 | <0.001 | 5.61 | 5.81 |
|  |  | Time (weeks) | 0.00 | 0.00 | <0.001 | 0.00 | 0.00 |
|  |  | Intervention Period | 0.02 | 0.01 | 0.041 | 0.00 | 0.04 |
|  | Retailer Affluence Group (Reference: Mid-Market) | Price Sensitive | -0.31 | 0.09 | 0.001 | -0.49 | -0.13 |
|  |  | Super Upmarket | 0.15 | 0.14 | 0.275 | -0.12 | 0.43 |
|  |  | Upmarket | 0.05 | 0.11 | 0.627 | -0.16 | 0.27 |
|  | Retailer Age Groups (Reference: Middle Aged) | Families | -0.11 | 0.10 | 0.278 | -0.30 | 0.09 |
|  |  | Retired | -0.37 | 0.23 | 0.114 | -0.82 | 0.09 |
|  |  | Young Adults | -0.01 | 0.08 | 0.845 | -0.16 | 0.13 |
|  |  | Intervention * Price Sensitive | 0.02 | 0.02 | 0.383 | -0.02 | 0.06 |
|  |  | Intervention * Super Upmarket | 0.10 | 0.03 | 0.003 | 0.04 | 0.17 |
|  |  | Intervention * Upmarket | 0.08 | 0.03 | 0.002 | 0.03 | 0.14 |
| Sales of meat (units) in Veganuary (with interaction terms) |  | Constant | 9.93 | 0.05 | <0.001 | 9.82 | 10.03 |
|  |  | Time (weeks) | 0.00 | 0.00 | 0.248 | 0.00 | 0.00 |
|  |  | Intervention Period | -0.01 | 0.01 | 0.369 | -0.04 | 0.01 |
|  | Retailer Affluence Group (Reference: Mid-Market) | Price Sensitive | -0.19 | 0.09 | 0.050 | -0.37 | 0.00 |
|  |  | Super Upmarket | 0.11 | 0.15 | 0.444 | -0.17 | 0.40 |
|  |  | Upmarket | 0.02 | 0.12 | 0.860 | -0.21 | 0.25 |
|  | Retailer Age Groups (Reference: Middle Aged) | Families | -0.07 | 0.10 | 0.503 | -0.27 | 0.13 |
|  |  | Retired | -0.32 | 0.24 | 0.191 | -0.79 | 0.16 |
|  |  | Young Adults | -0.09 | 0.08 | 0.252 | -0.25 | 0.06 |
|  |  | Intervention * Price Sensitive | 0.03 | 0.03 | 0.324 | -0.03 | 0.08 |
|  |  | Intervention * Super Upmarket | -0.05 | 0.04 | 0.223 | -0.14 | 0.03 |
|  |  | Intervention * Upmarket | 0.00 | 0.04 | 0.909 | -0.07 | 0.06 |
| Sales of plant-based dairy (units) in Veganuary (with interaction terms) |  | Constant | 5.47 | 0.05 | <0.001 | 5.36 | 5.58 |
|  |  | Time (weeks) | 0.01 | 0.00 | <0.001 | 0.01 | 0.01 |
|  |  | Intervention Period | -0.16 | 0.01 | <0.001 | -0.18 | -0.13 |
|  | Retailer Affluence Group (Reference: Mid-Market) | Price Sensitive | -0.40 | 0.10 | <0.001 | -0.59 | -0.21 |
|  |  | Super Upmarket | 0.31 | 0.15 | 0.034 | 0.02 | 0.60 |
|  |  | Upmarket | 0.11 | 0.12 | 0.326 | -0.11 | 0.34 |
|  | Retailer Age Groups (Reference: Middle Aged) | Families | -0.03 | 0.10 | 0.741 | -0.24 | 0.17 |
|  |  | Retired | -0.21 | 0.24 | 0.390 | -0.69 | 0.27 |
|  |  | Young Adults | 0.01 | 0.08 | 0.931 | -0.15 | 0.16 |
|  |  | Intervention * Price Sensitive | 0.07 | 0.03 | 0.022 | 0.01 | 0.12 |
|  |  | Intervention * Super Upmarket | 0.01 | 0.04 | 0.826 | -0.08 | 0.09 |
|  |  | Intervention * Upmarket | 0.02 | 0.03 | 0.545 | -0.05 | 0.09 |
| Sales of dairy (units) in Veganuary (with interaction terms) |  | Constant | 9.45 | 0.05 | <0.001 | 9.34 | 9.55 |
|  |  | Time (weeks) | 0.00 | 0.00 | <0.001 | 0.00 | 0.00 |
|  |  | Intervention Period | -0.02 | 0.01 | <0.001 | -0.04 | -0.01 |
|  | Retailer Affluence Group (Reference: Mid-Market) | Price Sensitive | -0.27 | 0.09 | 0.003 | -0.45 | -0.09 |
|  |  | Super Upmarket | 0.24 | 0.14 | 0.088 | -0.04 | 0.51 |
|  |  | Upmarket | 0.05 | 0.11 | 0.680 | -0.17 | 0.26 |
|  | Retailer Age Groups (Reference: Middle Aged) | Families | -0.05 | 0.10 | 0.616 | -0.24 | 0.14 |
|  |  | Retired | -0.17 | 0.23 | 0.452 | -0.63 | 0.28 |
|  |  | Young Adults | -0.03 | 0.08 | 0.671 | -0.18 | 0.12 |
|  |  | Intervention * Price Sensitive | 0.03 | 0.01 | 0.024 | 0.00 | 0.05 |
|  |  | Intervention * Super Upmarket | 0.00 | 0.02 | 0.835 | -0.03 | 0.04 |
|  |  | Intervention * Upmarket | 0.02 | 0.02 | 0.297 | -0.01 | 0.05 |

Supplementary Table 9. Interrupted time series models for the seasonal fruit intervention. Ratio models were run for both target and contextual fruit datasets. Difference models were run for promoted seasonal fruit products.

| **Model** | **Terms** | **Coefficient** | **Standard Error** | **p-value** | **Lower 95%** | **Upper 95%** |
| --- | --- | --- | --- | --- | --- | --- |
| Ratio  (£) of Sample A: Sample B (promoted products) | Constant | 0.506 | 0.002 | <0.001 | 0.503 | 0.509 |
|  | Time (weeks) | 0.000 | 0.000 | 0.866 | 0.000 | 0.000 |
|  | Intervention | 0.007 | 0.004 | 0.073 | -0.001 | 0.014 |
|  | Time*intervention | -0.001 | 0.000 | 0.002 | -0.002 | 0.000 |
| Ratio (units) of Sample A: Sample B (promoted products) | Constant | 0.505 | 0.001 | <0.001 | 0.502 | 0.508 |
|  | Time (weeks) | 0.000 | 0.000 | 0.950 | 0.000 | 0.000 |
|  | Intervention | 0.008 | 0.004 | 0.060 | 0.000 | 0.016 |
|  | Time*intervention | -0.001 | 0.000 | 0.003 | -0.002 | 0.000 |
| Ratio (£) of Sample A: Sample B (all products) | Constant | 1.043 | 0.002 | <0.001 | 1.040 | 1.046 |
|  | Time (weeks) | 0.000 | 0.000 | 0.067 | 0.000 | 0.000 |
|  | Intervention | -0.007 | 0.003 | 0.019 | -0.013 | -0.001 |
|  | Time*intervention | 0.000 | 0.000 | 0.264 | -0.001 | 0.000 |
| Ratio (units) of Sample A: Sample B (all products) | Constant | 1.048 | 0.002 | <0.001 | 1.044 | 1.052 |
|  | Time (weeks) | 0.000 | 0.000 | 0.589 | 0.000 | 0.000 |
|  | Intervention | -0.006 | 0.002 | 0.014 | -0.011 | -0.001 |
|  | Time*intervention | 0.000 | 0.000 | 0.249 | -0.001 | 0.000 |
| Difference  (in £) of Sample A from Sample B (promoted products) | Constant | 5225.794 | 143.164 | <0.001 | 4942.989 | 5508.599 |
|  | Time (weeks) | -6.157 | 1.768 | 0.001 | -9.650 | -2.665 |
|  | Seasonality (Fourier) | -1265.145 | 120.871 | <0.001 | -1503.913 | -1026.378 |
|  | Seasonality (Fourier) | 1138.698 | 108.950 | <0.001 | 923.480 | 1353.917 |
|  | Intervention | 850.930 | 242.474 | 0.001 | 371.950 | 1329.910 |
|  | Time*intervention | 16.477 | 33.040 | 0.619 | -48.789 | 81.744 |
| Difference (in units) of Sample A from Sample B (promoted products) | Constant | 2832.460 | 80.988 | <0.001 | 2672.477 | 2992.444 |
|  | Time (weeks) | -3.397 | 0.980 | 0.001 | -5.333 | -1.461 |
|  | Seasonality (Fourier) | -673.042 | 65.895 | <0.001 | -803.211 | -542.874 |
|  | Seasonality (Fourier) | 757.564 | 69.349 | <0.001 | 620.573 | 894.556 |
|  | Intervention | 802.086 | 286.907 | 0.006 | 235.334 | 1368.838 |
|  | Time*intervention | -23.304 | 35.173 | 0.509 | -92.784 | 46.176 |
| Difference (in £) of Sample A from Sample B (all products) | Constant | -102630.493 | 3514.723 | <0.001 | -109573.432 | -95687.555 |
|  | Time (weeks) | -46.005 | 41.714 | 0.272 | -128.405 | 36.396 |
|  | Seasonality (Fourier) | 3274.944 | 2962.883 | 0.271 | -2577.897 | 9127.785 |
|  | Seasonality (Fourier) | 9752.733 | 2332.134 | <0.001 | 5145.865 | 14359.600 |
|  | Intervention | 610.768 | 7464.722 | 0.935 | -14134.949 | 15356.484 |
|  | Time*intervention | 1260.514 | 718.496 | 0.081 | -158.794 | 2679.821 |
| Difference (in units) of Sample A from Sample B (all products) | Constant | -84897.052 | 2645.202 | <0.001 | -90122.349 | -79671.754 |
|  | Time (weeks) | 21.654 | 35.755 | 0.546 | -48.977 | 92.284 |
|  | Seasonality (Fourier) | -204.619 | 2343.792 | 0.931 | -4834.515 | 4425.277 |
|  | Seasonality (Fourier) | 8648.366 | 1602.450 | <0.001 | 5482.908 | 11813.825 |
|  | Intervention | -5196.687 | 4964.089 | 0.297 | -15002.685 | 4609.312 |
|  | Time*intervention | 935.919 | 328.021 | 0.005 | 287.951 | 1583.888 |

Supplementary Table 10. Multivariable hierarchical mixed-effects models for seasonal fruit intervention.

| **Model** |  | **Terms** | **Coefficient** | **Standard Error** | **p-value** | **Lower**  **95%** | **Upper**  **95%** |
| --- | --- | --- | --- | --- | --- | --- | --- |
| Promoted Fruit Sales (£) (during promotion period) |  | Intercept | 665.51 | 143.40 | <0.001 | 383.10 | 947.91 |
|  |  | Baseline Sales (£) | 1.25 | 0.02 | <0.001 | 1.21 | 1.30 |
|  | Sample (Reference: Sample A) | Sample B | 660.36 | 147.76 | <0.001 | 369.36 | 951.36 |
|  | Retailer Age Groups (Reference: Middle Aged) | Families | -17.62 | 135.13 | 0.896 | -283.73 | 248.50 |
|  |  | Retired | 728.44 | 370.82 | 0.051 | -1.85 | 1458.73 |
|  |  | Young Adults | 61.98 | 123.22 | 0.615 | -180.68 | 304.65 |
|  | Retailer Affluence Group (Reference: Mid-Market) | Price Sensitive | -62.76 | 150.68 | 0.677 | -359.51 | 233.99 |
|  |  | Super Upmarket | -193.27 | 200.68 | 0.337 | -588.49 | 201.95 |
|  |  | Upmarket | 23.17 | 143.08 | 0.872 | -258.62 | 304.96 |
| All Fruit Sales (£) (during promotion period) |  | Intercept | 20.12 | 3.02 | <0.001 | 14.16 | 26.07 |
|  |  | Baseline Sales (Rescaled) | 0.94 | 0.01 | <0.001 | 0.92 | 0.97 |
|  | Sample (Reference: Sample A) | Sample B | 1.06 | 1.64 | 0.519 | -2.17 | 4.30 |
|  | Retailer Age Groups (Reference: Middle Aged) | Families | -1.94 | 2.20 | 0.379 | -6.28 | 2.39 |
|  |  | Retired | 16.06 | 5.93 | 0.007 | 4.38 | 27.74 |
|  |  | Young Adults | -3.41 | 1.98 | 0.086 | -7.31 | 0.48 |
|  | Retailer Affluence Group (Reference: Mid-Market) | Price Sensitive | -0.91 | 2.38 | 0.702 | -5.60 | 3.78 |
|  |  | Super Upmarket | -8.81 | 3.18 | 0.006 | -15.08 | -2.55 |
|  |  | Upmarket | -3.03 | 2.26 | 0.183 | -7.49 | 1.43 |
| Promoted Fruit Sales (units) (during promotion period) |  | Intercept | 7.67 | 0.03 | <0.001 | 7.61 | 7.72 |
|  |  | Baseline Sales (Units) | 0.00 | 0.00 | <0.001 | 0.00 | 0.00 |
|  | Sample (Reference: Sample A) | Sample B | 0.15 | 0.03 | <0.001 | 0.10 | 0.21 |
|  | Retailer Age Groups (Reference: Middle Aged) | Families | 0.03 | 0.03 | 0.206 | -0.02 | 0.09 |
|  |  | Retired | 0.04 | 0.07 | 0.622 | -0.11 | 0.18 |
|  |  | Young Adults | 0.04 | 0.02 | 0.101 | -0.01 | 0.09 |
|  | Retailer Affluence Group (Reference: Mid-Market) | Price Sensitive | -0.06 | 0.03 | 0.053 | -0.12 | 0.00 |
|  |  | Super Upmarket | 0.01 | 0.04 | 0.896 | -0.07 | 0.08 |
|  |  | Upmarket | 0.04 | 0.03 | 0.188 | -0.02 | 0.09 |
| All Fruit Sales (units) (during promotion period) |  | Intercept | 4.17 | 0.02 | <0.001 | 4.12 | 4.22 |
|  |  | Baseline Sales (Units) Rescaled | 0.01 | 0.00 | <0.001 | 0.01 | 0.01 |
|  | Sample (Reference: Sample A) | Sample B | 0.00 | 0.01 | 0.988 | -0.03 | 0.03 |
|  | Retailer Age Groups (Reference: Middle Aged) | Families | 0.00 | 0.02 | 0.912 | -0.04 | 0.03 |
|  |  | Retired | 0.10 | 0.05 | 0.035 | 0.01 | 0.19 |
|  |  | Young Adults | 0.01 | 0.02 | 0.687 | -0.02 | 0.04 |
|  | Retailer Affluence Group (Reference: Mid-Market) | Price Sensitive | -0.03 | 0.02 | 0.121 | -0.07 | 0.01 |
|  |  | Super Upmarket | -0.07 | 0.02 | 0.008 | -0.11 | -0.02 |
|  |  | Upmarket | -0.02 | 0.02 | 0.294 | -0.05 | 0.02 |
