# Supplementary material for: Do promotions of healthier or more sustainable foods increase sales? Findings from three natural experiments in UK supermarkets
Source: BMC Public Health. 2024 Jun 21;24:1658. doi: 10.1186/s12889-024-19080-x (PMC11191299; doi:10.1186/s12889-024-19080-x)
Supplement: Supplementary file 2 — Supplementary Material 2. [file 12889_2024_19080_MOESM2_ESM.docx]

**Appendix**

*No added sugar (NAS) plant-based milk*

1. The following code was used to identify products for the entire product list of plant-based milks for inclusion in the NAS plant-based milk analysis:

grepl("unsweetened|no added sugar|no sugars|Alpro Light Fresh Drink Alternative|ALPRO SOYA LIGHT", plantbasedmilk_df$product, ignore.case = TRUE)

*Veganuary*

1. The following code was used to identify promoted products that were of particular interest during the Veganuary intervention (i.e. products that were plant-based substitutes rather than items such as canned vegetables, etc.):

grepl("alpro|beyond|birds eye|bol|bosh|c/nut|califia|cauldron|cheese alt|chicken free|

coconut collaborative|dairy free|falafel|[brand exclusive to supermarket]|fresh drink alt|fresh milk alt|

meat free|free from|ginsters|goodfellas|goodlife|gosh|gu|heck|higgidy|linda|magnum vegan|

Mrs Crimbles|naked|oumph|pizza express|plantastic|pukka vegan|quorn|richmond 8 meat free|

smoky ham free|squeaky bean|strong roots|[supermarket own plant-based brand name]|meatless farm|tofoo|vegetarian butcher|

plant-based|this isn't bacon|vegan|vegan factor|violife|vivera|[brand exclusive to supermarket]|salsa pizza

|ice cream|kirsty|meat free|meat-free|ham free|alt", veganuary_df$product, ignore.case = TRUE)

1. The following code was used to exclude items erroneously included in the dairy category:

grepl("(?i)BRUSCHETTA|bread|croissant|dough|pizza", dairy_df$product)

1. The following code was then applied to identify dairy products for analysis:

grepl("(?i)ice cream|mozzarella|dough ice|peace pop|extra thick cream", dairy_df$product)

1. The following code was used to identify the non-dairy products for analysis:

grepl("(?i)alpro|dairy free|plant|almond milk|soy milk|coconut milk|vegan|

almond breeze|oat milk|cheese alt|oatly|oat drink|vegetable fat|

veg fat|plant-based|plnt/bsd|alpro|andros|soy|non-dairy|

non dairy|dairy-free|dairy free|almond|alternative|califia|coconute collaborative|good hemp|koko|coconut drink|oat drink|little coco nutters|nurishh|oatly|[supermarket brand name] free from|almond drink|soya drink|violife|vitalite||[brand exclusive to supermarket]|olive spread|pure sunflower", dairy_df$product)

1. Meat and plant-based meat categories were identified by retailer classification (buyer category), as well as the following code to identify items that may have been classified incorrectly as meat:

grepl("(?i)quorn|plant|veg|meat free|free from meat", pm.meat$description)
